# Developmental transcriptomic patterns can be altered by transgenic overexpression of Uty

**DOI:** 10.1038/s41598-023-47977-x

**Published:** 2023-11-30

**Authors:** Kylie D. Rock, Lillian M. Folts, Hannah C. Zierden, Ruth Marx-Rattner, Nicolae Adrian Leu, Bridget M. Nugent, Tracy L. Bale

**Affiliations:** 1 Center for Epigenetic Research in Child Health and Brain Development, University of Maryland School of Medicine, Baltimore, USA; 2 Department of Pharmacology, University of Maryland School of Medicine, Baltimore, MD 21201 USA; 3 Present Address: Department of Biological Sciences, Clemson University, Clemson, SC 29634 USA; 4 Present Address: Biomedical Sciences Graduate Program, University of Colorado Anschutz Medical Campus, Aurora, CO 80045 USA; 5 Present Address: Department of Chemical and Biomolecular Engineering, University of Maryland, College Park, MD 20740 USA; 6 Department of Biomedical Sciences, School of Veterinary Medicine, University of Pennsylvania, Philadelphia, PA 19104 USA; 7 University of Colorado School of Medicine, CU Anschutz Medical Campus, 12800 E. 19th Avenue, Aurora, CO 80045 USA; 8 Present Address: Department of Psychiatry, University of Colorado Anschutz Medical Campus, Aurora, CO 80045 USA

**Keywords:** Developmental biology, Neuroscience

## Abstract

The genetic material encoded on X and Y chromosomes provides the foundation by which biological sex differences are established. Epigenetic regulators expressed on these sex chromosomes, including *Kdm6a* (*Utx*), *Kdm5c*, and *Ddx3x* have far-reaching impacts on transcriptional control of phenotypic sex differences. Although the functionality of UTY (*Kdm6c*, the Y-linked homologue of UTX), has been supported by more recent studies, its role in developmental sex differences is not understood. Here we test the hypothesis that UTY is an important transcriptional regulator during development that could contribute to sex-specific phenotypes and disease risks across the lifespan. We generated a random insertion *Uty* transgenic mouse (Uty-Tg) to overexpress *Uty*. By comparing transcriptomic profiles in developmental tissues, placenta and hypothalamus, we assessed potential UTY functional activity, comparing *Uty*-expressing female mice (XX + Uty) with wild-type male (XY) and female (XX) mice. To determine if *Uty* expression altered physiological or behavioral outcomes, adult mice were phenotypically examined. *Uty* expression masculinized female gene expression patterns in both the placenta and hypothalamus. Gene ontology (GO) and gene set enrichment analysis (GSEA) consistently identified pathways including immune and synaptic signaling as biological processes associated with UTY. Interestingly, adult females expressing *Uty* gained less weight and had a greater glucose tolerance compared to wild-type male and female mice when provided a high-fat diet. Utilizing a *Uty*-overexpressing transgenic mouse, our results provide novel evidence as to a functional transcriptional role for UTY in developing tissues, and a foundation to build on its prospective capacity to influence sex-specific developmental and health outcomes.

## Introduction

Sex differences in physiology, morphology, and behavior exist across the lifespan and contribute to significant differences in disease risk and resilience^1^. Established in early development, these differences including primary sex determination (i.e., gonadal) arise as the product of sex hormones and sex chromosomes where, in most cases, females are XX and males are XY^2^. Genes expressed on the X and Y chromosomes undoubtedly play a significant role in the timing and progression of sex-specific development, especially for the brain^2–6^. How specific genes, especially on the Y chromosome, impart sex-specific differences in tissue development and function is still largely unknown. Many X- and Y-linked genes are important in broadly controlling transcriptional responses critical to normal developmental processes^7,8^. However, the sex specificity to such regulatory mechanisms is less understood.

**Figure 1:**
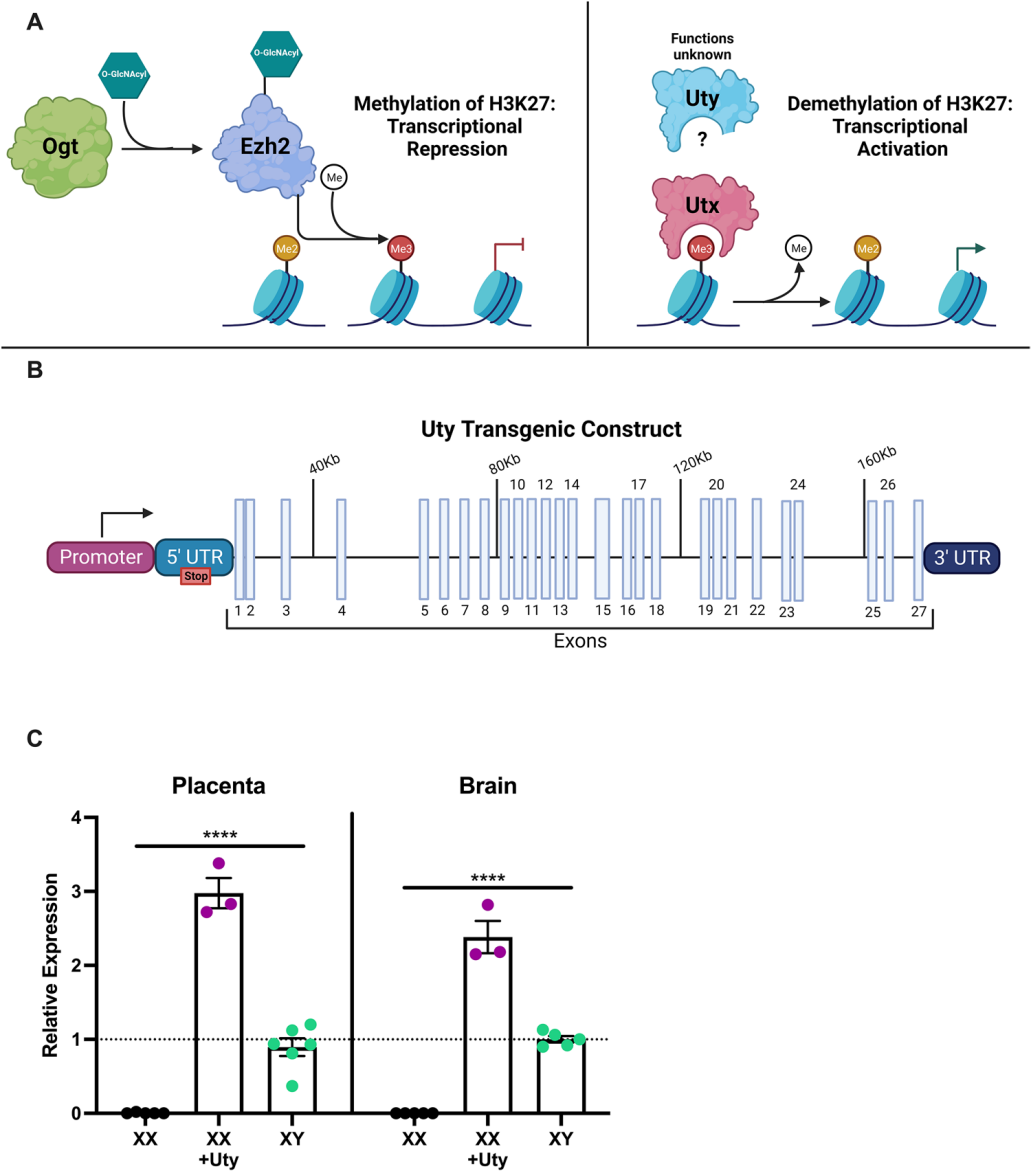
Uty transgenic construct and expression levels during development in the placenta and brain. (**A**) Schematic of enzymes involved in methylation (glycosylated EZH2) and demethylation (UTX and potentially UTY) of histone 3 lysine 27. (**B**) Simplified schematic of *Uty* transgenic construct. See Supplementary File 1 for more details. (**C**) Uty mRNA expression in E18.5 placenta and brain from XX females, XX + *Uty* females, XY males (n = 3–6). A significant effect of genotype was observed for relative mRNA expression in the placenta (F (2,11) = 135.4, p = 0.0001; η^2^ = 0.96, ****p ≤ 0.0001; **C**), with XX + Uty females having significantly higher *Uty* expression compared to XX females and XY males (p ≤ 0.0001; p ≤ 0.0001) and XY males having significantly higher *Uty* expression compared to XX females (p = 0.0003). In the hypothalamus a significant effect of genotype was observed for mRNA expression (F(2,10) = 165.2, p = 0.0001; η^2^ = 0.97, ****p ≤ 0.0001; **C**), with XX + Uty females having significantly higher *Uty* expression compared to XX females and XY males (p ≤ 0.0001; p ≤ 0.0001) and XY males having significantly higher *Uty* expression compared to XX females (p ≤ 0.0001). (**A,B**) Were created with www.BioRender.com.

Ubiquitously transcribed X chromosome tetratricopeptide repeat protein, or UTX, is a widely expressed X-linked demethylase^9–12^. UTX demethylates di- and tri-methylated histone 3 lysine 27 (H3K27me2/3), promoting a euchromatin state or active state of transcription (Fig. 1A)^13,14^. Few studies have probed the biological function of UTY, the Y-linked homologue of UTX. Unlike UTX, current in vitro and in vivo evidence suggests that UTY lacks demethylase activity due to missing sequence within its catalytic domain^11,14,15^. However, recent studies provide evidence that UTX and UTY do share some functional redundancy, refuting the assumption that UTY is a nonfunctional remnant of UTX, and play an important role in differentiation, development, and pathophysiology, including cancer and heart disease^14,16–26^. For example, male *Utx* knock-out (KO) mouse embryos express normal levels of *Uty* and survive until birth, similar to heterozygous *Utx* females, while homozygous *Utx*-KO is embryonically lethal to females, suggesting UTY is functionally compensating for some aspects required for development in the absence of UTX in males. Further interrogation of UTY function in vitro previously demonstrated that redundancy in function did not appear to be specific to H3K27 demethylation, but rather was associated with other proteins and protein complexes that broadly regulate transcription^14,16^. Interestingly, discordance also exists in brain *Utx* and *Uty* expression patterns, where male mice showed much higher expression of *Uty* than *Utx* in the hypothalamus, suggesting a potential novel role for UTY in neuroendocrine regulation^27^. Still, there remains a paucity of data as to our understanding of functional roles of UTY, especially during fetal development in tissues where sex-specific regulation would be important, such as the placenta and hypothalamus.

Recent studies from our lab and others have demonstrated that sex differences in transcriptional regulation in the placenta can be partially attributed to differential expression of the X-linked gene O-linked *N*-acetylglucosamine transferase (*Ogt*) between males and females^28–31^. Furthermore, sex differences in OGT dependent regulation of the H3K27 methyltransferase enhancer of zeste homologue 2 (EZH2) contributes to female resilience to the programmatic effects of prenatal stress (Fig. 1A)^29,30,32^. However, the contribution of Y-linked genes to dissimilarities in placental transcriptional regulation has yet to be explored and may provide novel insight as to the gestational mechanisms conferring male-specific disease risk. Here we test the hypothesis that UTY is involved in regulation of male-specific gene expression using the placenta and hypothalamus as important developmental tissues with known sex differences in form and function. To examine the potential role of UTY, we developed a transgenic mouse with a random insertion of the full *Uty* gene (Uty-Tg; Fig. 1B). An unbiased transcriptomic approach was used as a proxy for UTY function by comparing female mice with *Uty* expression (XX + Uty) to wild-type male (XY) and female (XX) mice, utilizing transcriptomics tools for a conceptual methodological examination for broad gene expression changes, e.g., GSEA, as evidence for UTY function^33–37^. To probe changes in UTY demethylase activity in our transgenic mice, we utilized our previously published H3K27me3 chromatin immunoprecipitation sequencing (ChIP-Seq) dataset to assess overlap in UTY gene regulation with male-specific H3K27me3 binding to examine the potential role of UTY in transcriptional repression^32^. Finally, we examined adult phenotypic outcomes relevant to alterations in hypothalamic function, including the HPA stress axis and feeding and body weight gain in response to a calorically dense dietary challenge.

## Methods

### Animals

All experiments were approved by the University of Maryland School of Medicine and the University of Pennsylvania Institutional Animal Care and Use Committees and performed in accordance with the National Institutes of Health Animal Care and Use Guidelines. Reporting of animal experiments is done in accordance with ARRIVE guidelines. The recombinant *Uty* DNA construct (180 kb) was generated by Gen-H, Genetic Recombineering Heidelberg, using a BAC clone backbone, floxed STOP cassette in the 5ʹ UTR, and the entire *Uty* gene, including the 1.35 kb of the endogenous promoter localized 20 kb upstream of the transcriptional start site and all 27 exons (Supplemental File 1 Figs. 1–6). Transformation, selection, and injection of embryonic stem cells carrying this linear DNA construct into the inner cell mass of C57BL/B6 mouse blastocysts was performed at the School of Veterinary Medicine, University of Pennsylvania to establish the Uty transgenic strain (Uty-Tg). All Uty-Tg mice were derived from successful random integration into a single clone, which we acknowledge is a limitation of this study. However, the absence of profound changes to the transcriptome under control conditions as shown and discussed below, suggest that our results were not significantly impacted by the initial BAC integration. While a conditional Uty-overexpressing mouse was our original intended approach, in our examination of *Uty* tissue overexpression, we unfortunately confirmed a lack of tissue specificity and thus we are assessing outcomes as a global *Uty*-overexpressing mouse (Supplemental File 1 Fig. 7). Relative expression of *Uty* mRNA was validated in the E18.5 XX + Uty fetal placenta and brain and demonstrated that Uty-Tg animals express *Uty* at levels that were equal to or greater than wild-type male endogenous expression levels (i.e., *Uty*-overexpressing; Fig. 1C). Our goal was also to assess UTY protein expression, however, we were unable to find an antibody that was specific to UTY and clearly distinct from UTX to probe changes in protein expression.

Mice were housed under a 12 h light/day photoperiod with lights on at 0700 EST and ad libitum access to water and grain-based chow diet (Purina Rodent Chow 5001, St. Louis, MO). Dams (50:50 C57BL/B6:129-CYP19-Cre, PCre) were paired with (C57BL/B6-Uty-Tg) sires to generate offspring that were on a B6:129 background. It was not until after we had already bred our animals on a PCre background that we discovered *Uty* overexpression was, in fact, not conditional. Therefore, we moved forward with assessing transcriptomic changes in tissues that had been collected that were on the same B6:129 background and did not express PCre, to be consistent. For fetal collections dams and sires were paired overnight, starting at 1700 and separated at 0700 EST and checked for a copulation plug^38^. Noon on the day that the plug was observed was considered embryonic day 0.5 (E0.5). Dams were euthanized at E18.5 using a precision vaporizer with an induction chamber and waste gas scavenger in which isoflurane was administered in 2.5% O_2_ for 1 min followed by rapid decapitation. Fetal collections took place at E18.5 and tissues were collected for genotyping and RNA-sequencing (Supplemental File 1 Fig. 8). Fetal tissues used for sequencing were all first generation on a B6:129 background and did not express Pcre (Supplemental File 1 Fig. 8). Several primer combinations were tested identifying the primers P587-check1 (5ʹ-CACTGGTGATGACGCAAGTC-3ʹ) and GBPR281 (5ʹ-CACCACTGCTCCCATTCATC-3ʹ) as the optimal combination for consistent Uty-Tg genotyping (Supplemental File 2). Embryonic sex determination was achieved by genotyping using primers specific for Jarid1 (5ʹ-TGAAGCTTTTGGCTTGAG-3ʹ and 5ʹ-CCGCTGCCAAATTCTTTGG-3ʹ) as previously described^39^. For offspring phenotypic assessments, dams were allowed to litter, and pups remained with their mothers undisturbed until weaning at postnatal day 28 (PN28).

### Tissue collection

For fetal collections, pregnant dams were euthanized at E18.5 and fetal tails, heads, and placentas were collected. DNA was isolated from fetal tails for genotyping and sexing. One XY male, one XX female, and one XX + Uty female were used for each analysis to eliminate potential confounding litter effects. Fetal heads and hemisected (i.e., halved) placentas were placed in cryotubes, and frozen in liquid nitrogen. Tissues were stored at − 80 °C until RNA isolation.

### RNA sequencing and analysis

E18.5 fetal heads were cryosectioned at 300 µm, − 20 °C on a Cryostat. Using a 1 mm tissue punch, two 1 mm × 300 µm thick punches were collected that corresponded to images 63–68 in the P0 section of the Atlas of the Developing Mouse Brain to obtain a punch enriched for the paraventricular nucleus (PVN) of the hypothalamus, referred to as the hypothalamus throughout the manuscript^40^. Micropunches were immediately dispensed into 500 µL of Trizol and stored at – 80 °C until RNA isolation. Placentas were homogenized in 500 µL of Trizol in preparation for RNA extraction and purification. For both the hypothalamus and placenta, RNA was extracted from tissue/Trizol homogenates via chloroform liquid–liquid extraction and alcohol precipitation. Purification of mRNA was performed using Qiagen RNeasy Mini kits and Illumina cDNA libraries of E18.5 hypothalamus and placental mRNA were prepared from 25 and 300 ng total RNA, respectively, using the Illumina Stranded mRNA Prep kits (20040532, Illumina) and indexes (20040553, Illumina) according to the manufacturer’s protocol. Library fragment size was quantified using Agilent High Sensitivity D1000 ScreenTape Assays (5067–5584, Agilent). Library concentrations were determined using the Qubit dsDNA High Sensitivity Assay (Q32851, ThermoFisher). Samples were multiplexed and sequenced on an Illumina NextSeq550 instrument using high output 1 × 75 bp chemistry (20024906, Illumina).

FASTQ files generated from Illumina were concatenated and used as an input to kallisto, a pseudoalignment program, for alignment to the *Mus musculus* reference transcriptome (version 38)^41^. The remaining analysis took place in the R statistical environment (Version 4.1.1; R Core Team 2021). Gene isoforms were assigned to gene symbols using the Bioconductor package tximport and genes were filtered to counts per million > 1 in at least four samples for the hypothalamus and > 1 in at least five for placental analysis^42^. One wild-type female sample was removed from the hypothalamic analysis due to an issue with library preparation. The filtered gene lists were then normalized using trimmed mean of M-values in edgeR (Supplemental File 1 Figs. 9, 10), variance weights were calculated using voom, and differential expression analysis of linear fit models was performed using limma, Benjamini-Hochburg false discovery rate (FDR) < 0.1 and log fold change (logFC) > 1^43–45^.

Because there are transcription variants of *Uty* alternative splicing (AS) analysis was performed. Hisat2 v.2.2.1 was used as a splice-aware aligner to create the genome index for Mus musculus GRCm38 (release 79) reference genome^46^. The bam files obtained after the corresponding mapping were inputted in rMATS v.4.1.2 to identify splicing events^47^. These events were then utilized to determine if the different *Uty* variants identified corresponded to protein coding transcripts. Bedops v2.4.41 toolset was used to create a BED file from the genome annotation and use it as a template to map the reads to each annotated transcript^48^. MultiBamCov from the Bedtools toolkit v. 2.29.2 was used to count the alignments per transcript in each input bam file and filter the counts that are spanning the protein coding transcripts^49^.

As we did not observe significant differences in individual genes based on conservative corrections for multiple comparisons, adjusted p ≤ 0.05, we utilized gene set enrichment analysis (GSEA) as we hypothesized that expression of *Uty* would likely have a significant overall impact on expression of numerous genes and biological processes, as is the function of a GSEA method. GSEA (GSEAv4.3.2, Broad Institute, Cambridge, MA) of normalized counts were used to identify patterns of gene expression and determine greater-than-chance enrichment of biological pathways in a threshold-free manner (i.e. without consideration for differential gene expression), as previously reported by our work and that of other labs^33,35,50^. Briefly, collections of GO subcategory biological processes (GO:BP), molecular function (GO:MF), and cellular component (GO:CC) annotated gene sets were obtained from the Molecular Signature Database (MSigDBv7.4.1, Broad Institute, Cambridge, MA) available for use with GSEA software. Gene set permutations were computed in GSEA to determine FDR, nominal p value, and normalized enrichment score (NES) of each gene set with a significance threshold set at FDR < 0.05 and NES > 1.

As a supplemental hypothesis-generating approach, we also utilized previously published transcriptomic and ChIP-seq data sets to assess a role for UTY in sex-specific gene expression and transcriptional regulation. In this study, we identified modules in our heatmaps with differential gene expression patterns that were directionally similar (i.e., similarly upregulated or downregulated based on z-score) for XX + Uty females and XY males but different from XX females that we refer to as masculinized XX + Uty gene expression. We compiled a list of 229 placental genes and 182 hypothalamic genes that showed significant differences, p ≤ 0.05, in expression between XX females and XY males from these published datasets to compare to our masculinized XX + Uty genes^32,51^. Finally, we integrated our previously published H3K27me3 ChIP-Seq dataset from the placenta with our masculinized XX + Uty placental genes^32^.

### Phenotypes

To communicate the practical significance (i.e., magnitude of effect), effect sizes were calculated for all phenotypic measures. ANOVA effect size was determined by calculating Eta squared (η^2^), effects of which are defined as small at 0.01, medium at 0.06, and large at 0.14. Effect sizes for post-hoc Tukey’s or Sidak’s corrections for multiple comparisons were calculated by Cohen’s d, effects of which are defined as small at 0.2, medium at 0.5, and large at 0.81^52^.

### Animal weights

For all studies in adult animals, first generation offspring on a B6:129 mixed background received a unique identification ear tag at PN28 and were weighed weekly from PN28–PN70. At PN70, animals were placed on a high-fat diet (HFD; Research Diets Inc, D12492), calories provided by protein 29% and fat 14%, or remained on the grain-based control chow diet (Purina Rodent Chow 5001, St. Louis, MO), calories provided by protein 20% and fat 60%, to assess phenotypic outcomes following a 5-week dietary challenge. Two-way repeated measures and one-way ANOVAs, with Sidak’s or Tukey’s multiple comparisons tests, respectively, were conducted using Prism (Graphpad), α = 0.05.

### Corticosterone response to restraint stress

PN56 XX, XY, and XX + Uty offspring underwent restraint stress by being placed in a 50 mL conical tube for 15 min. A single < 1 mm distal tail snip was made at time 0 to collect 10 µL of tail blood at 0, 15, 30, and 120 min, which was placed in EDTA-treated tubes. Quantification of plasma corticosterone levels was determined by radioimmunoassay (207,120, MP Biomedicals). Two-way repeated measures ANOVA with Sidak’s multiple comparisons post-hoc test was conducted using Prism (Graphpad), α = 0.05.

### Food consumption

Food consumption was assessed at PN84 for a 24 h period by weighing food at hour 0 and again at hour 24. Animals were pair-housed in the same genotype pairs for this assessment and data was analyzed as weight of food consumed per animal, assuming each animal ate the same amount of food, and normalized to individual body weight. A one-way ANOVA with Tukey’s multiple comparisons post-hoc test was conducted using Prism (Graphpad), α = 0.05.

### Glucose tolerance test

A glucose tolerance test was administered at PN91 to XX, XY, and XX + Uty offspring. Animals were fasted for 6 h before receiving an intraperitoneal injection of 0.3 g/mL glucose in saline. A Contour Next Blood Glucose Monitoring System (Bayer Co, Germany) was used to measure blood glucose levels. A single < 1 mm distal tail snip was made to collect tail blood at 0, 30, 60, and 120 min from the time of injection. Two-way repeated measures ANOVA with Sidak’s multiple comparisons post-hoc test was conducted using Prism (Graphpad), α = 0.05.

## Results

### Random insertion of the Uty-Tg increases relative mRNA expression of Uty and yields full-length Uty transcripts in the E18.5 placenta and hypothalamus

A significant effect of genotype was observed for relative mRNA expression in the placenta (F (2,11) = 135.4, p = 0.0001; η^2^ = 0.96; Fig. 1C), with XX + Uty females having significantly higher *Uty* expression compared to XX females and XY males (p ≤ 0.0001; p ≤ 0.0001) and XY males having significantly higher *Uty* expression compared to XX females (p = 0.0003). Similarly, in the hypothalamus a significant effect of genotype was observed for relative mRNA expression (F(2,10) = 165.2, p = 0.0001; η^2^ = 0.97; Fig. 1C), with XX + Uty females having significantly higher *Uty* expression compared to XX females and XY males (p ≤ 0.0001; p ≤ 0.0001) and XY males having significantly higher *Uty* expression compared to XX females (p ≤ 0.0001). The transcripts produced by our Uty-Tg approach corresponded to protein coding sequences that contain all 27 exons, suggesting upregulation of a full-length functional *UTY* protein (Supplemental Data 1, Tables S1, S2).

### Placenta and hypothalamic gene expression

**Figure 2:**
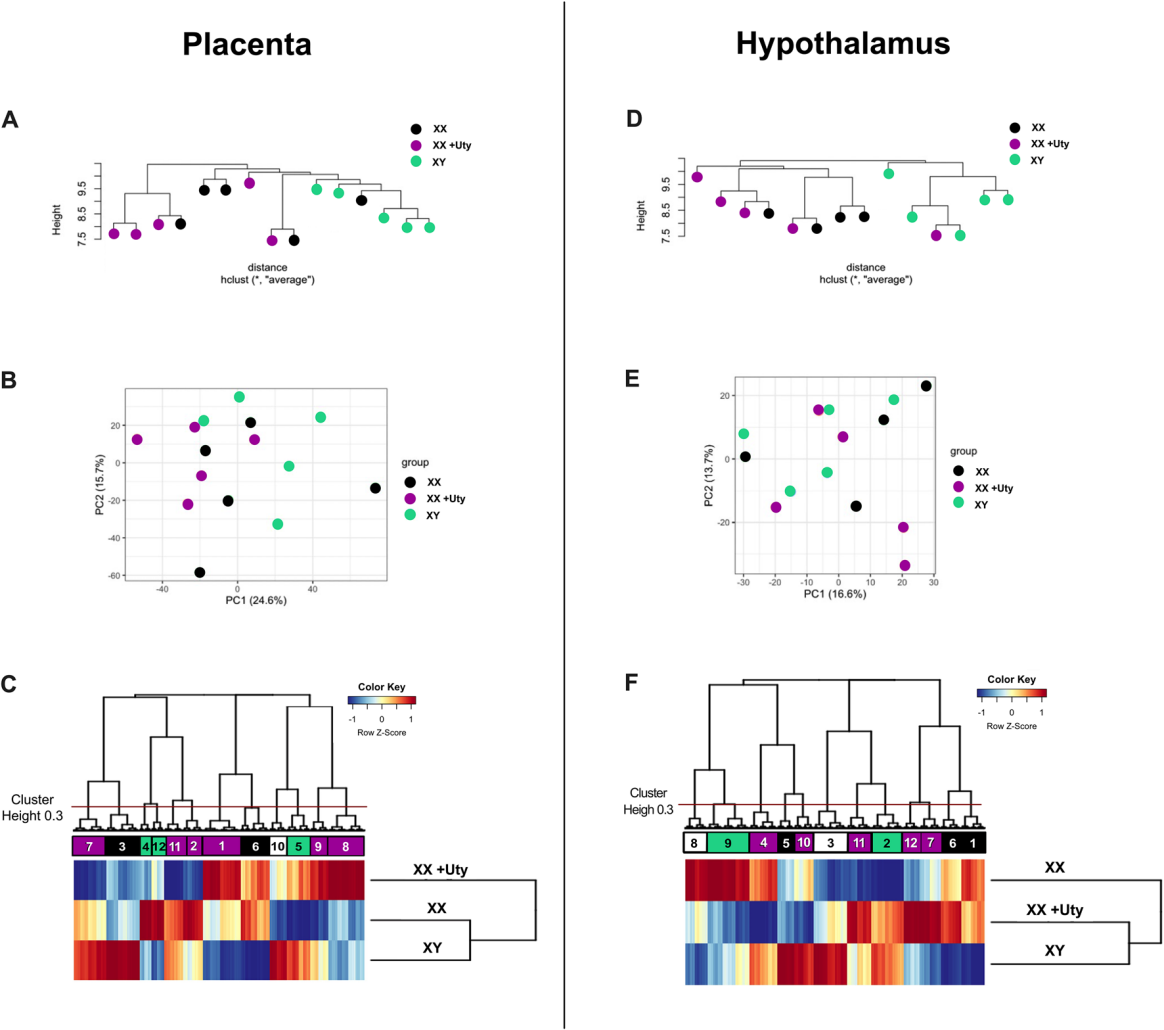
Global changes in gene expression were not observed with random insertion of Uty-Tg in XX females. (**A**) Cluster dendrogram and (**B**) principal component analysis (PCA) plots of gene expression data demonstrate the distribution of XY, XX, and XX + Uty placenta (n = 5). (**C**) Heatmap depicting all detectable transcripts in the placenta (14,230). Unbiased hierarchical clustering was used to group genes into distinct clusters and identify gene sets that were similar in directionality of their expression profiles between XX + Uty and XX (black), XX + Uty and XY (green, masculinized), differential expression unique to XX + Uty (purple), or expression profiles that did not show a unique or overlapping pattern between the groups (white). (**D**) The same approach and figures as above are also provided for all detectable transcripts in the hypothalamus cluster dendrogram, (**E**) PCA plot, and (**C**) heatmap of gene expression data demonstrating the distribution of XY, XX, and XX + Uty hypothalamic patterns (n = 4–5).

Cluster dendrograms and principal components analysis (PCA) showed little separation between XX + Uty and XX females in the placenta (Fig. 2A,B) or hypothalamus (Fig. 2D,E). Differential gene expression analysis (contrast matrix XX + Uty female − XX female; FDR < 0.1, LogFC > 1) revealed no significant changes in individual gene expression, with the exception of *Uty*, between the genotypes in the placenta or hypothalamus. Additionally, expression of *Utx* was not altered by overexpression of *Uty* in XX + Uty animals (Supplemental File 1 Fig. 11). Collectively, these findings suggest that changes in overall gene expression in XX + Uty animals compared to XX did not result from our random insertion overexpressing transgenic approach. However, as a random insertion method does not allow for confirmation of where or how many insertions in the genome have occurred, we acknowledge this potential methodological confound.

Hierarchical clustering was performed on all detectable transcripts without p-value or logFC restrictions (p = 1, logFC = 0) to look at broad differences in gene expression profiles between XX + Uty, XX, and XY animals. In the placenta, there were a total of 14,230 detectable transcripts that were used for hierarchical clustering and grouped into twelve distinct modules. We identified modules with differential gene expression patterns that were directionally similar (i.e., similarly upregulated or downregulated based on z-score) for XX + Uty compared to XX females, similar for XX + Uty females and XY males compared to XX females, as well as modules with differential expression that was unique to XX + Uty (e.g., upregulated in XX + Uty but downregulated in XX and XY), or expression profiles that did not show a change in directionality of regulation (Fig. 2C). Modules 4, 5, and 12 are referred to as masculinized because the direction of change in XX + Uty females relative to XX females is the same as the direction of change in XY males relative to XX females.

In the hypothalamus, the total number of detectable transcripts was 14,553, which were used for hierarchical clustering and grouped into twelve distinct modules. Similar to the placenta, we identified modules with differential gene expression patterns that were directionally similar for XX + Uty compared to XX females, similar for XX + Uty females and XY males compared to XX females, as well as modules with differential expression that was unique to XX + Uty (e.g., upregulated in XX + Uty but downregulated in XX and XY), or expression profiles that did not show a change in directionality of regulation (Fig. 2F). Modules 9 and 2 are referred to as masculinized because the direction of change in XX + Uty females relative to XX females is the same as the direction of change in XY males relative to XX females.

### Transcripts in XX + Uty mice are significantly enriched for biological processes including immune response and synaptic signaling in the placenta and hypothalamus, respectively

**Figure 3:**
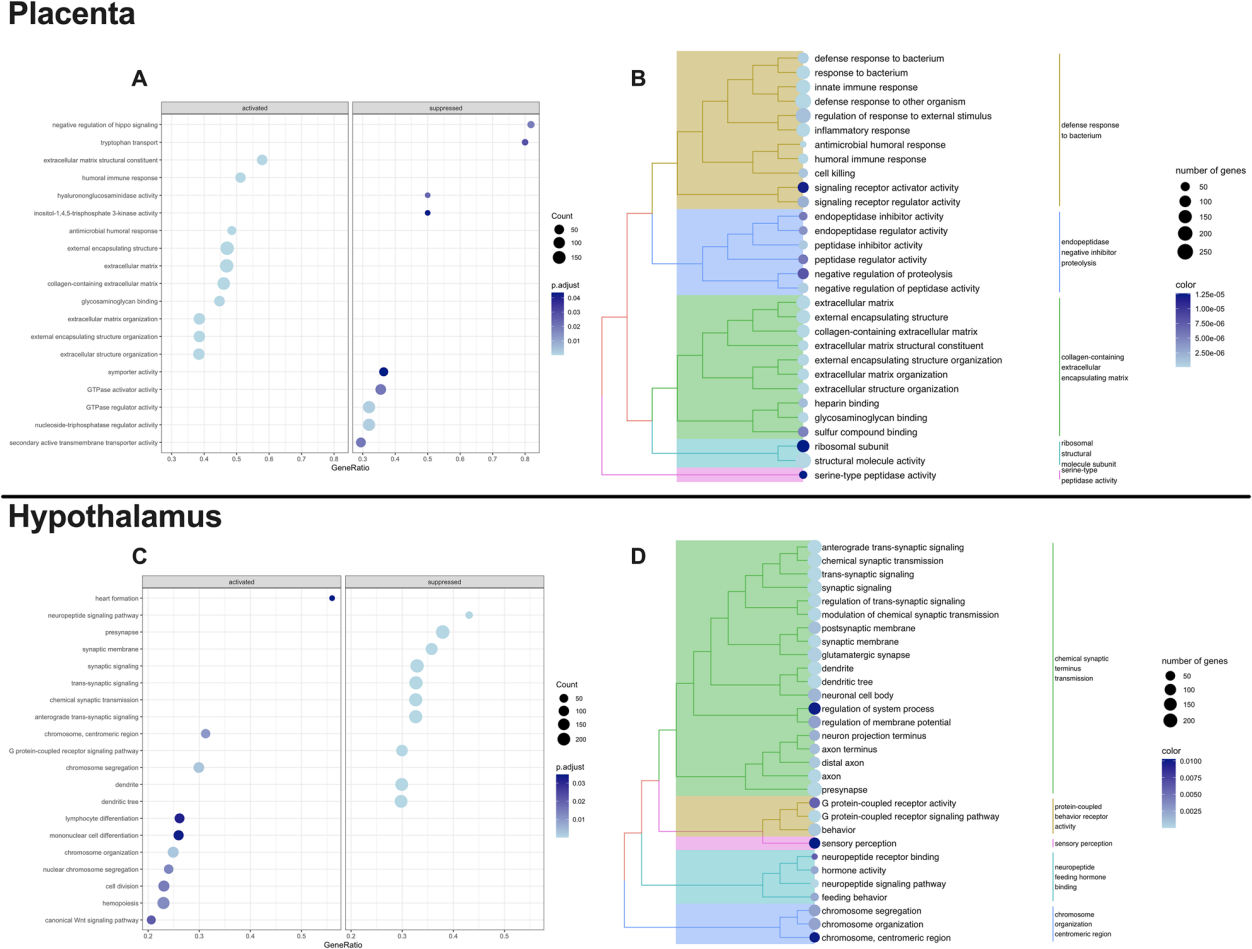
XX + Uty animals show gene set enrichment for biological processes associated with immune response in the placenta and synaptic signaling in the hypothalamus compared to XX mice. Placenta (n = 5). (**A**) Dot plot depicting biological processes identified by gene set enrichment analysis (GSEA). Bubble color indicates adjusted p-value (p.adjust) and bubble diameter represents gene set size. Plots are split into activated (left panel) and suppressed (right panel) biological processes and are plotted in order of gene ratio (fraction of genes in dataset belonging to an ontology over total number genes in an ontology). (**B**) Tree plot depicting hierarchical clustering of GSEA enriched terms. Bubble color indicates adjusted p-value (p.adjust) and bubble diameter represents gene set size. Hypothalamus (n = 4–5): The same approach and figures as above are provided for all detectable transcripts in the hypothalamus. (**C**) dot plot depicting biological processes identified by GSEA, (**D**) tree plot depicting hierarchical clustering of GSEA enriched terms.

Tissue-specific GSEA analysis of normalized counts was performed, with FDR < 0.05, NES > 0 ~ XX + Uty phenotype, and NES < 0 ~ XX female phenotype (Supplemental Data 1, Tables S3, S4). Dot plots represent the top 20 significantly enriched biological processes, separated into activated or suppressed processes. In the XX + Uty placenta, biological processes including tryptophan transport, hyaluronidase activity, and negative regulation of hippo signaling were suppressed while humoral immune response, extracellular matrix, and extracellular structural organization were activated compared to XX placentas (Fig. 3A). Using pairwise similarities of the enriched terms, hierarchical clustering of enriched biological processes was performed to aid in interpretation of biological significance for enriched pathways. In the XX + Uty placenta, the enriched pathways clustered into five different groups, including defense response to bacterium, endopeptidase negative inhibitor proteolysis, collagen-containing extracellular encapsulating matrix, ribosomal structure/molecule subunit serine-type, and peptidase activity (Fig. 3B).

In the hypothalamus, GSEA was performed as described for the placenta analysis above. In the XX + Uty hypothalamus, biological processes including neuropeptide signaling, synaptic signaling, and dendrites were suppressed while chromosome segregation, lymphocyte differentiation, and cell division were activated compared to XX hypothalamus (Fig. 3C). Furthermore, hierarchical clustering of enriched pathways identified similar pathways enriched in the XX + Uty phenotype and grouped them into chemical synaptic terminus transmission, protein-coupled receptor activity, sensory perception, neuropeptide feeding hormone binding, and chromosome organization centromeric region (Fig. 3D).

### Masculinized gene expression patterns and transcriptional regulation in the XX + Uty female placenta and hypothalamus

**Figure 4:**
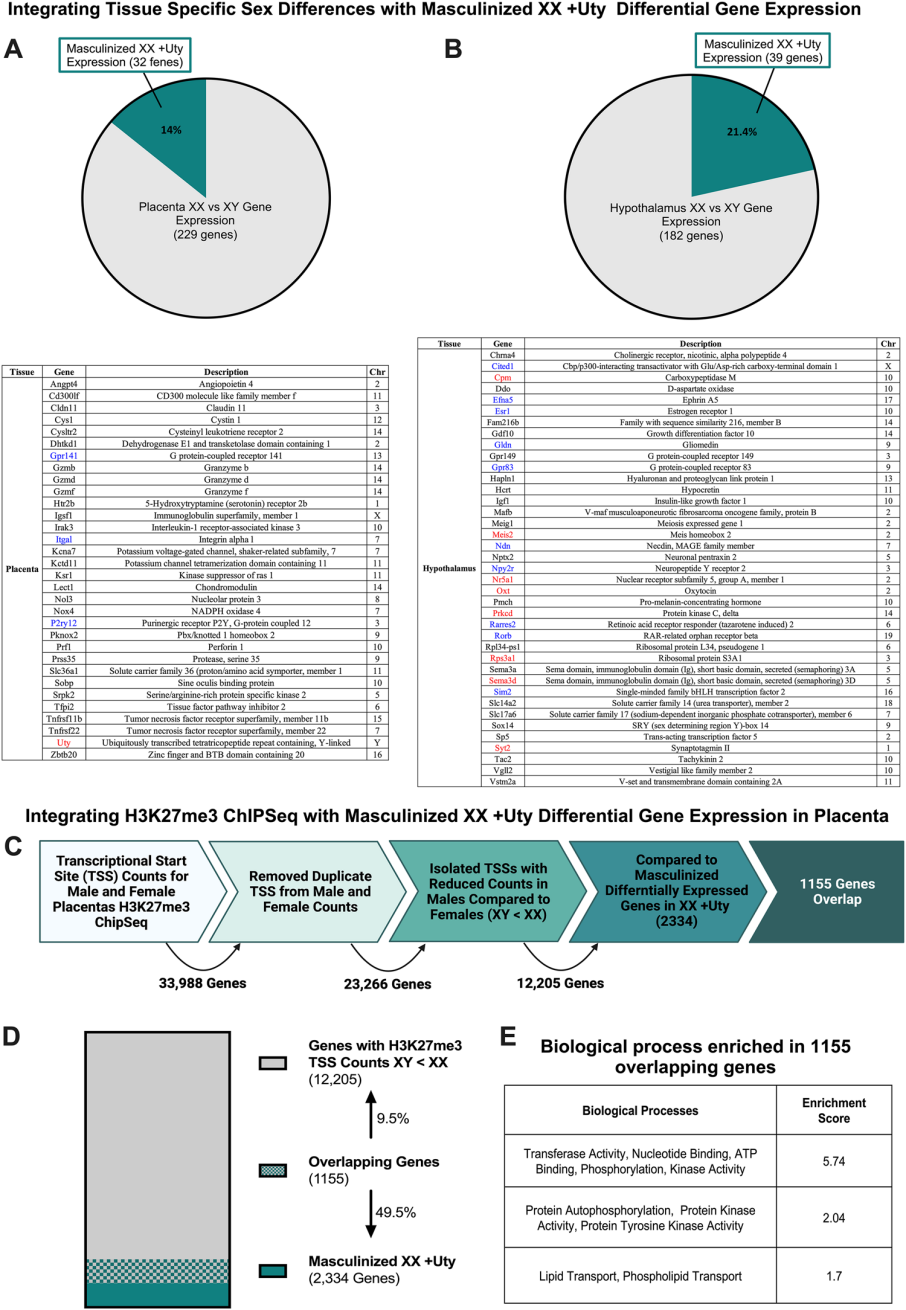
Masculinized XX + Uty genes are shared between tissues, with established sex differences, and with genes that have male specific H3K27me3 profiles. Pie charts depicting overlap in masculinized XX + Uty and previously identified and published sex differences in the (**A**) placenta (n = 5) and (**B**) hypothalamus (n = 4–5)^32,51^. Gene names, descriptions, and chromosomes on which they are located are detailed in the inserted table, with genes that were similarly upregulated (red) or downregulated (blue) indicated by the colored text. (**C**) Schematic describing how previously published H3K27me3 ChIP-Seq data from male placentas was used and compared to genes that were masculinized in the XX + Uty gene set^32^. (**D**) Parts of a whole bar plot depicting the proportion of genes with TSS counts XY < XX (gray, 12,205 genes) and masculinized XX + Uty genes (green, 2334) that overlap (gray and green patterned, 1155 genes). Overlapping genes represent 9.5% of the genes identified in the male H3K27me3 ChIP-Seq and 49.5% of the XX + Uty masculinized dataset. (**E**) Biological processes enriched in the 1155 overlapping genes, identified by DAVID functional annotations.

As a supplement, we utilized previously published data sets to assess a role for UTY in sex-specific gene expression and transcriptional regulation. Focusing on genes that showed male-like differential expression patterns in XX + Uty tissues, we compared our masculinized XX + Uty genes to previously reported sex differences in gene expression in the hypothalamus and placenta (Fig. 4A,B)^32,51^. Of interest and developmentally important, we identified estrogen receptor alpha (*Esr1*) and several estrogen responsive genes, oxytocin (*Oxt*), insulin-like growth factor 1 (*Igf1*), and protein kinase C delta (*Prkcd*), in both our masculinized XX + Uty gene list and reported sex differences in the hypothalamus, suggesting a potential interaction between UTY and estrogen receptor transcriptional regulation^53–57^.

Finally, we integrated our previously published H3K27me3 chromatin immunoprecipitation sequencing (ChIP-Seq) dataset from the placenta of mice on a B6:129 background with our masculinized XX + Uty female placental genes^32^. The ability of UTY to act as a demethylase, similar to its X-linked homologue, UTX, has yet to be determined. To test the hypothesis that the presence of UTX and UTY in male (XY) cells would contribute to increased H3K27me3 demethylation and therefore fewer H3K27me3 marks at transcriptional start sites, we first filtered the ChIP-Seq data accordingly. This process involved identifying genes with reduced H3K27me3 TSS counts in males (XY) compared to females (XX) and then comparing the list of genes to our masculinized XX + Uty gene list (Fig. 4C). Notably, we identified 1155 genes shared between the filtered ChIP-Seq dataset and the masculinized XX + Uty gene list, resulting in 9.5% of all genes we had previously identified with H3K27me3 TSS counts where XY < XX and 49.5% of the masculinized XX + Uty gene list (Fig. 4D). Pathway analysis of these masculinized genes highlight processes including transferase activity, phosphorylation, and lipid transport as significantly enriched (Fig. 4E).

It is critical to emphasize that these approaches utilized differential gene expression data with unadjusted p ≤ 0.05, therefore caution is warranted when interpreting these results. However, it is also important to highlight these comparisons and findings as useful starting points for future investigations into the role of UTY in tissue- and sex-specific gene expression.

### Uty confers vulnerability to metabolic dysregulation in HFD females

**Figure 5:**
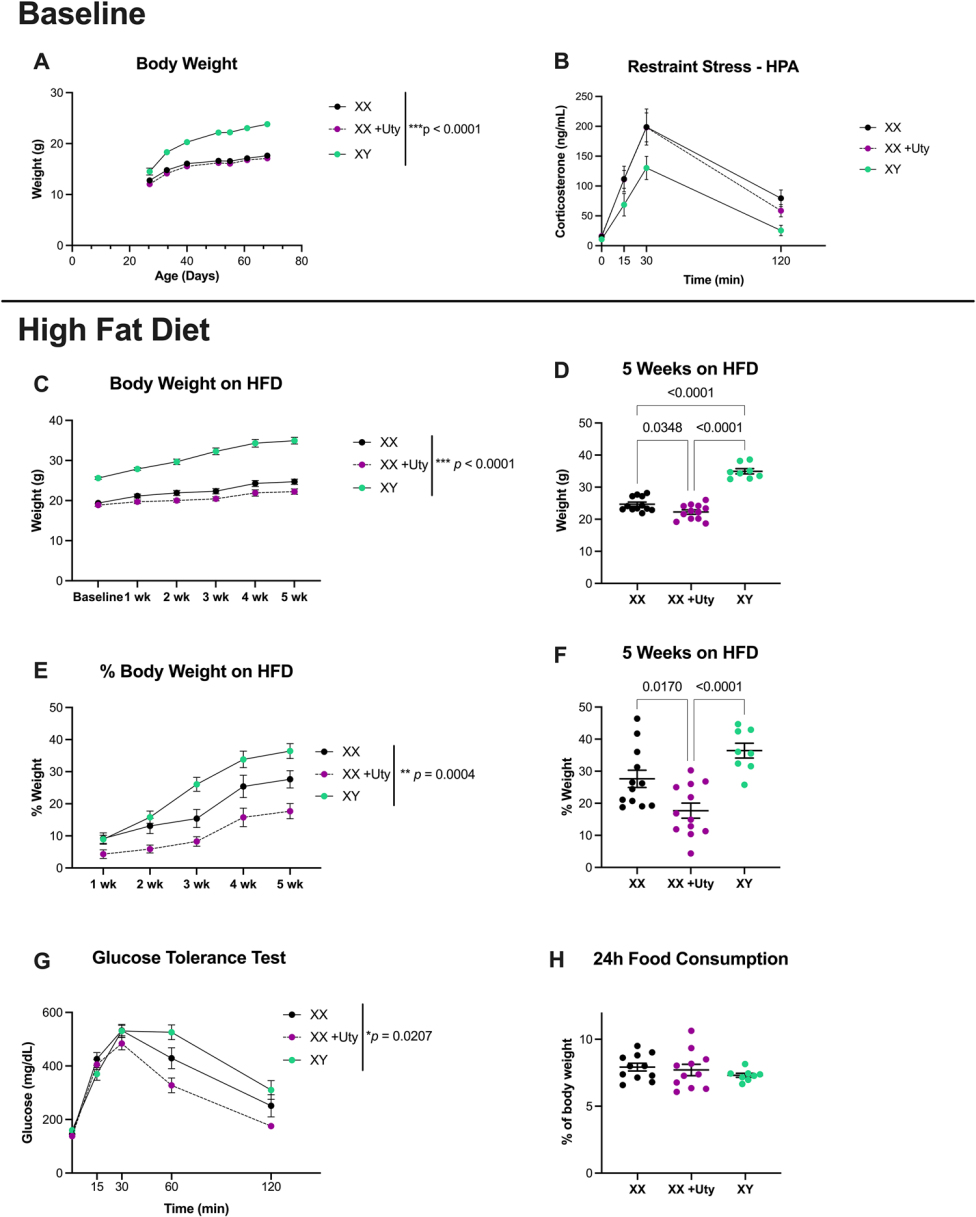
XX + Uty animals gain less weight and have a higher glucose tolerance when challenged with a high fat diet compared to XX and XY mice. (**A**) Body weights were collected weekly from PN28–70. A significant effect of genotype was observed for baseline body weight measurements (F (2,29) = 63.4, p = 0.0001; η^2^ = 0.81), with XY being consistently heavier than XX + Uty (PN28, p = 0.02; PN35–PN70, p ≤ 0.0001; d = 1.98) and XX (PN35, p = 0.0002; PN42–70, p ≤ 0.0001; d = 1.81). There was no significant effect of Uty-Tg on XX + Uty bodyweight (n = 8–12). (**B**) Regulation of corticosterone levels time course was assessed following a 15-min restraint stress with sampling at 0, 15, 30, and 120 min. No significant effect of Uty-Tg was observed on XX hypothalamic–pituitary–adrenal-axis (HPA) response (n = 7–13). Following baseline assessments, mice were challenged with a high fat diet (HFD) for 5 weeks. Body weights were collected weekly while on the diet. (**C**) A significant effect of genotype was observed throughout the 5-week dietary challenge (F (2,29 = 85.08, p = 0.0001; η^2^ = 0.85), where XY mice were heavier than XX + Uty (Wk1–5, p ≤ 0.0001; d = 3.70) and XX mice (Wk1–5, p ≤ 0.0001; d = 2.86). (**D**) At the end of the 5-week HFD challenge, there was still a significant effect of genotype (F(2,29) = 79.50, p = 0.0001; η^2^ = 0.85, with XX mice weighing less than XY mice (p ≤ 0.0001; d = 4.52) and XX + Uty weighing less than XY and XX mice (p ≤ 0.0001; d = 5.50 and p = 0.03; d = 1.08). (**E**) A significant effect of genotype was observed for percent weight gained on HFD over the 5-week period (F (2,29) = 10.45, p = 0.0004; η^2^ = 0.42), with XX + Uty consistently having a lower percent weight gain than XY mice (Wk2, p = 0.01; Wk3, p = 0.0002; Wk4, p = 0.003; Wk5, p = 0.0004; d = 1.50). (**F**) This significant effect of genotype persisted to the end of the five-wk dietary challenge (F (2,29) = 12.65, p = 0.0001; η^2^ = 0.47), where XX + Uty mice had a significantly lower percent weight change than XY and XX mice (p = 0.02; d = 2.54; p ≤ 0.0001; d = 1.14). (**G**) Glucose tolerance test (GTT) was performed at 0, 15-, 30-, 60-, and 120-min. Genotype had a significant effect on glucose tolerance (F (2,31) = 4.407, p = 0.02; η^2^ = 0.22), with XX + Uty mice having significantly lower glucose levels at 60 and 120 min compared to XY mice (p = 0.0002; p ≤ 0.0001; d = 0.48). (**H**) Food consumption per cage (n = 4–5 cages with 2 mice/cage) was measured by weighing food at 0 and 24 h, and showed a significant effect of genotype (F(2,11) = 11.87, p = 0.0018; η^2^ = 0.68) with XY males consuming significantly more food compared to XX and XX + Uty females (p = 0.02; d = 2.08; p = 0.0014; d = 4.89).

A significant effect of genotype was observed for baseline body weight measurements (F (2,29) = 63.4, p = 0.0001; η^2^ = 0.81; Fig. 5A), with males consistently heavier than XX + Uty (PN28, p = 0.02; PN35–PN70, p ≤ 0.0001; d = 1.98) and XX females (PN35, p = 0.0002; PN42–70, p ≤ 0.0001; d = 1.81). No significant effect of Uty-Tg was observed for baseline female body weight measurements (Fig. 5A) or male body weights (Supplemental File 1 Fig. 12). The fact that XY and XY + Uty body weights did not differ suggests that there may not be a gene dosage effect on metabolic outcomes, however more work is needed to thoroughly investigate distinct gene dosage impacts. No main effect of genotype was observed for stress response measured by restraint stress and HPA activity (Fig. 5B), however post-hoc analysis did reveal that XY males had significantly lower levels of corticosterone at 120 min compared to XX and XX + Uty females (p = 0.007; d = 0.65; p = 0.04; d = 0.56). When animals were challenged with a HFD, a significant effect of genotype was observed (F (2,29 = 85.08, p = 0.0001; η^2^ = 0.85; Fig. 5C), where males were heavier than XX + Uty (Wk1–5, p ≤ 0.0001; d = 3.70; Fig. 5C) and XX females (Wk1–5, p ≤ 0.0001; d = 2.86; Fig. 5C). Body weight assessments at the end of the 5 week dietary challenge revealed a significant effect of genotype (F(2,29) = 79.50, p = 0.0001; η^2^ = 0.85; Fig. 5D), with XX females weighing less than XY males (p ≤ 0.0001; d = 4.52; Fig. 5D) and XX + Uty females weighing less than XY males and XX females (p ≤ 0.0001; d = 5.50 and p = 0.03; d = 1.08; Fig. 5D). A significant effect of genotype was observed for percent weight gained on HFD over the 5-week period (F (2,29) = 10.45, p = 0.0004; η^2^ = 0.42; Fig. 5E), with XX + Uty females consistently having a lower percent weight gained than XY males (Wk2, p = 0.01; Wk3, p = 0.0002; Wk4, p = 0.003; Wk5, p = 0.0004; d = 1.50; Fig. 5E). This significant effect of genotype persisted to the end of the 5-week dietary challenge (F (2,29) = 12.65, p = 0.0001; η^2^ = 0.47; Fig. 5F), where XX + Uty females had a significantly lower percent weight change than XY males and XX females (p = 0.02; d = 2.54; p ≤ 0.0001; d = 1.14; Fig. 5F). To assess if these changes in body weight corresponded to changes in glucose regulation and food consumption, we ran a glucose tolerance test (GTT) and measured the 24-h food consumption. Genotype had a significant effect on glucose tolerance (F (2,31) = 4.407, p = 0.02; η^2^ = 0.22; Fig. 5G), with XX + Uty females having significantly lower glucose levels at 60 and 120 min compared to XY males (p = 0.0002; p ≤ 0.0001; d = 0.48; Fig. 5G). Finally, a significant effect of genotype was observed for 24-h food consumption (F(2,11) = 11.87, p = 0.0018; η^2^ = 0.68) with XY males consuming significantly more food compared to XX (p = 0.02; d = 2.08) and XX + Uty females (p = 0.0014; d = 4.89; Fig. 5H).

## Discussion

Sex differences in fetal development and sensitivity to intrauterine perturbations are well documented, however, there remains a paucity of information on specific molecular and/or genetic mechanisms responsible for divergent male and female development and health outcomes. We previously showed that OGT, a protein encoded by an X-linked gene, contributes to female fetal protection from prenatal stress, in part through its regulation of the H3K27 methyltransferase, EZH2^29,30,32^. Increased OGT is associated with higher levels of H3K27me3 in the female placenta, in both mouse and human tissue^32^. However, less is understood as to the sex-specific roles of the X- and Y-linked H3K27 demethylases, UTX and UTY. Comparisons of UTX and UTY amino acid sequences demonstrate extensive sequence homology, reaching 88% homology, both within and outside of the catalytic domain, yet the function of UTY remains less clear^14,15^. Important to sex-differences in H3K27me3-mediated transcriptional regulation in the placenta, XY tissues naturally have reduced OGT in addition to the presence of UTY, potentially culminating in overall reduced levels of H3K27me3 and reduced transcriptional repressive control in tissues such as the placenta^32^. As such, we hypothesized that these differences may account for an increased vulnerability for males in utero, especially for neurodevelopmental risk^4–6,58^. We previously reported no sex difference for placental *Utx* expression suggesting that *Utx*, unlike *Ogt*, may not escape X inactivation in the placenta^29,32^. Therefore, we hypothesized that if UTY indeed had transcriptional influence, either via potential demethylation of H3K27me3 and/or other as of yet unidentified activity, it would serve as a molecular mechanism that could direct male-specific fetal development. Therefore, we generated a *Uty*-overexpressing transgenic mouse and utilized a transcriptomic approach to assess potential changes in placental and fetal brain development.

Initial phenotypic assessment found no overt developmental changes or outcomes in the XX + Uty mice. As *Uty* is not a master regulator (i.e., not known to be at the top of a gene regulation hierarchy) and limited protein–protein interactions have been described for UTY, we did not anticipate that over-expression of *Uty* in a wild-type female mouse would produce profound phenotypic changes. We examined global health outcomes in the XX + Uty mice and confirmed that they showed no signs of developmental abnormalities or health consequences resulting from *Uty* expression. Significant differences in gene expression based on conservative corrections for multiple comparisons and distinct differences in clustering assessed by cluster dendrogram and principal components analysis were not observed in the placenta or hypothalamus. We interpret these findings as an indication that we have not caused significant, potentially detrimental changes to the genome by the random insertion approach. Furthermore, the lack of baseline changes in body weight and functionality of the stress-axis (HPA) in XX + Uty compared to XX animals indicates that growth, metabolism, and neuroendocrine regulation were relatively normal, under control housing and feeding conditions.

In the placenta, our GSEA identified pathways related to biological processes, molecular functions, and cellular components including inflammatory/immune response and extracellular matrix genes. Regulation of immune tolerance is essential for successful pregnancy and relies on coordination and communication between neighboring cells. The semi-allogeneic placenta derives half of its genetic material from the mother and half from the fetus (maternal and paternal) that leads to the expression of foreign paternal proteins in the intrauterine environment^59,60^. Therefore, several mechanisms are in place to protect the placenta from being targeted by the maternal immune system^61^. The observed enrichment in pathways associated with inflammatory and immune responses in placentas with *Uty* expression raises interesting questions regarding the role of UTY in chronic placental inflammation, a phenotype more often observed with male fetuses and associated with premature delivery^62,63^. Other studies have reported associations between experimentally reduced *Uty* expression and changes in immune response, although there are inconsistencies in the directionality of immune dysregulation^64,65^.

The extracellular matrix also plays an important role in the placenta, providing structural support and aiding in trophoblast invasion of the uterus^66^. Tissue stiffness, largely controlled by the composition of the extracellular matrix, has been established as an important regulator of cellular processes, in both health and disease^67–69^, and has recently been shown to vary in placental tissue across patients diagnosed with pregnancy complications, such as preeclampsia and intra-uterine growth restriction^70–73^. Not much is known about how or if UTY modulates the extracellular matrix, although there is some suggestive evidence that UTY plays a role in male-specific susceptibility to atherosclerosis and modulates gene expression related to atherosclerosis, including extracellular matrix genes^65^. A better understanding of the potential role of UTY in regulating the expression of extracellular matrix genes could shed light on the molecular origins of sex differences in risk of numerous pathologies, including adverse pregnancy outcomes.

In the hypothalamus, key processes including synaptic transmission and neuropeptide/hormone activity were enriched with *Uty* expression. More specifically, pathways related to synaptic transmission, including the pre-synapse, synaptic membrane, and dendrite were suppressed in the XX + Uty compared to XX hypothalamus. This finding is surprisingly similar to recent reports in the hippocampus demonstrating that *Utx* also modulates genes involved in dendritic morphology and synaptic transmission, results that suggest a role for UTX and UTY in synaptic development and plasticity^74^. Given the sex and brain region-specific differences in expression of *Utx* and *Uty*, a regulatory role in synaptic development may contribute to sex differences in form and function across brain regions^27^. Enrichment and hierarchical clustering of neuropeptide signaling, hormone activity, and feeding behavior pathways suggests that growth, metabolism, and neuroendocrine regulation, key hypothalamic functions may be modulated by UTY. Evidence of overlapping functional roles for UTX and UTY in both metabolism and development has recently been reviewed and supports our transcriptomic findings in the developing XX + Uty hypothalamus^13^.

In examination of adult phenotypes for effects of UTY, while adult XX + Uty females showed no significant differences from XX females under normal control conditions, they presented with surprising alterations in body weight and glucose tolerance following a 5-week calorically dense dietary challenge. While we hypothesized that a detectable phenotype would resemble wild-type males, we observed that XX + Uty females did not present in a masculinized manner. Surprisingly, XX + Uty females at the end of the HFD exposure weighed less and had a *greater* glucose clearance compared to either wild-type males or females. As this phenotype was unique to XX + Uty females, we interpret this finding as a potential role for UTY in metabolic regulation that may be related to developmental changes or current adult activity of UTY and may be distinct from XY and XX mice as a result of conflicting or compounding effects of UTY on an XX background. In examination of food consumption as an explanation for body weight differences, we found that XX + Uty females in fact consumed fewer calories than males on the HFD. However, no significant differences were detected between XX + Uty females and XX females suggesting again unique interactions of UTY on an XX background that are impacting metabolic processes. Combined with our transcriptomic data, particularly the hypothalamic enrichment for neuropeptide signaling, hormone activity, and feeding behavior, these data highlight UTY as a potential molecular target of metabolic programming during development and phenotypic outcome in adulthood. Future investigations into the mechanisms and hormonal regulation driving this *Uty*-associated phenotype could provide important insight about sex differences in metabolism and risk of metabolic disorders.

## Conclusions and limitations

Epigenetic regulators expressed from X and Y chromosomes have significant potential to impart broad sex differences in transcriptional regulation, and to contribute to sex-specific responses to environmental perturbations. In such studies, the functional role of UTY has largely been ignored, however, the few studies that do exist suggest that it retains biological functions that may be independent from H3K27me3 demethylation^14,15^. We specifically focused on the placenta and hypothalamus for this study due to the plethora of evidence showing that these tissues respond to changes in the intrauterine environment in a sex-specific manner. While our transcriptomic analysis was tissue-specific, our expression of *Uty* was not. Therefore, an important limitation of our study is regarding the global overexpression of *Uty* and the broad changes in any tissue that could produce secondary effects in the placenta or brain, and vice versa. Moving forward it would be important to modify the *Uty* construct to conditionally express the transgene. Furthermore, the random insertion of transgenes rather than a targeted insertion approach can disrupt normal gene sequences and yield unpredictable phenotypes and is a potential confounding factor in our study. We recognize the importance of utilizing site-specific overexpression and knockout models in future studies to probe if deletion of *Uty* results in loss of a proposed functions in XY males. Another limitation of the current studies is that we focused on examining the addition of the Uty transgene on a XX background to assess the role of Uty in potential masculinizing sex difference effects on transcription. We did not examine the potential role of gene dosage of Uty (adding Uty to XY). As developmental impacts at the cellular level of XX vs XY cells influence relevant outcomes, it is likely that these are interesting but distinct questions that can be addressed in future studies. We did note that XY + Uty mice did not differ in the body weight trajectories from XY mice, suggesting interesting regulatory complexities to dosage effects of Uty that we did not examine here (Supplemental File 1 Fig. 12). It is also important to note that in tissues where UTX escapes X-chromosome inactivation, such as the brain, it has been postulated that the UTX-UTY pair serves as a dosage-sensitive regulator. Therefore, it is possible that the observed differences in XX + Uty mice could be attributed to increased expression of the UTX-UTY pair, rather than specific function of UTY^75^. More work is needed to further characterize the molecular functions and contributions to phenotypic outcomes by UTY. In conclusion, these studies provide novel insight as to the potential role of UTY in gene expression that may underlie sex differences in risk and resilience across the lifespan and provide a foundation to build on in future studies.

## Supplementary Information

Supplementary Information.

Supplementary Tables.

## Data Availability

The RNA-seq data generated in this study has been deposited in the NCBI SRA database under accession code PRJNA883432 (http://www.ncbi.nlm.nih.gov/bioproject/883432). All other raw data are available upon request.
