# Supplementary material for: Developmental transcriptomic patterns can be altered by transgenic overexpression of Uty
Source: Sci Rep. 2023 Nov 30;13:21082. doi: 10.1038/s41598-023-47977-x (PMC10687263; doi:10.1038/s41598-023-47977-x)
Supplement: Supplementary file 1 — Supplementary Information. [file 41598_2023_47977_MOESM1_ESM.docx]

**Supplemental Methods**

To generate a conditional *Uty*-overexpressing mouse, a construct was designed where a loxP-flanked stop cassette was inserted into the *Uty* construct. Creation and characterization of the BAC clone was performed by Gen-H, Genetic Recombineering Heidelberg (**Supplemental Figure 1 – 6**). Crossing Uty-Tg animals with transgenic animals carrying tissue specific Cre-recombinase expression would produce tissue-specific Uty-overexpression. We bred our Uty-Tg males with C57BL/B6:129-CYP19-Cre (PCre) females or Tg(Sim1-cre)1Lowl/J (Sim1Cre) females and identified animals there were positive for the Uty-Tg using the primers P587-check1 (‘5-CACTGGTGATGACGCAAGTC-3’) and GBPR281 (‘5-CACCACTGCTCCCATTCATC-3’) as the optimal combination for consistent Uty-Tg genotyping (**Supplemental Figure 6**). Using quantitative real-time polymerase chain reaction (qRT-PCR) we compared the relative abundance of *Uty* mRNA in the placenta (PCre, **Supplemental** **Figure 7A**) and hypothalamus (Sim1Cre), respectively. Unfortunately, we noted a lack of tissue-specific correspondence for *Uty* expression in mice from a Uty x Sim1Cre background indicating that our stop cassette was not functioning as anticipated leading to global overexpression of *Uty* (**Supplemental Figure 7B**). Similarly, we found overexpression of *Uty* in Uty x PCre tissue across life stages. Elevated *Uty* expression was observed in the cerebellum of XY *+*Uty males and females as well as XX +Uty +PCre females (**Supplemental Figure 7C**). General distribution of RNA-Seq gene expression data was visualized using scatter and violin plots, demonstrating consistency across replicates in both the placenta and hypothalamus (**Supplemental Figure 9A – 9D and 10A – 10D)**. Assessment of counts per million gene expression data show that in the absence of Cre Uty-Tg animals over express *Uty* without altering the expression of *Utx* (**Supplemental Figure 11**).


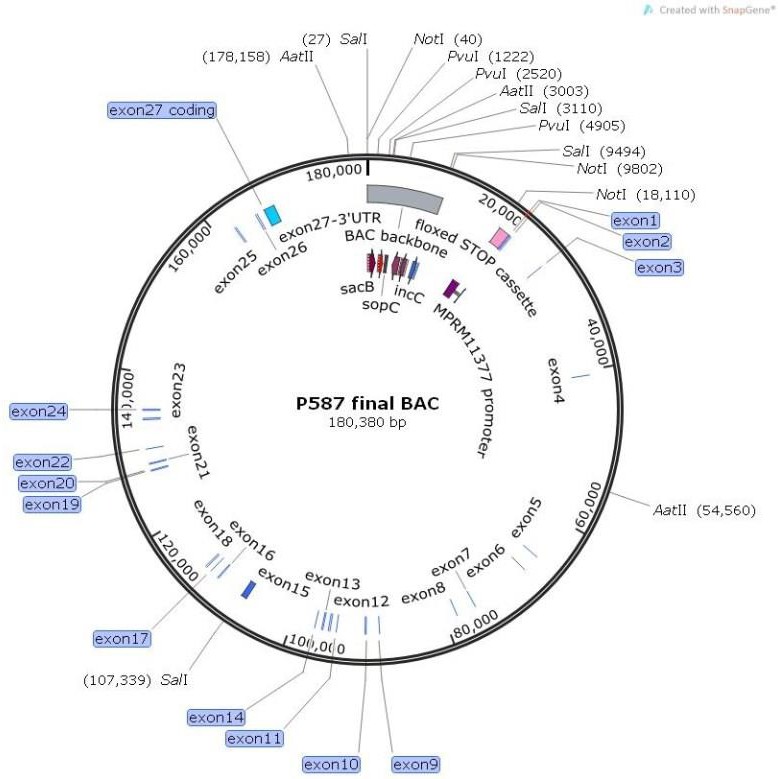


**Supplemental Figure 1:** Map of the final BAC clone (bMQ-300b04; floxedSTOP; backbone-loxP removed); functional parts are indicated.


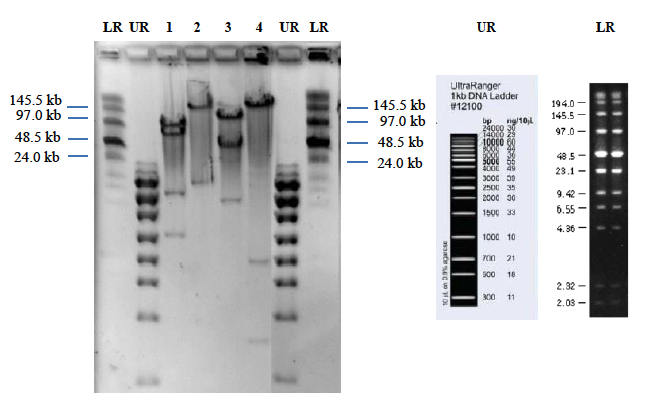


**Supplemental Figure 2:** Restriction digestion of the final modified BAC clone; BAC DNA was digested either with *Sal*I (lane 1), *Not*I (lane 2), *Aat*II (lane 3) or *Pvu*I (lane 4). The restriction fragments were separated by pulsed-field gel electrophoresis (PFGE). UR = UltraRanger 1kb DNA ladder; LR = LowRange PFG marker. Expected fragments for *Sal*I (97.845 bp + 73.068 bp + 6.384 bp + 3.083 bp), *Not*I (162.310 bp + 9.762 bp + 8.308 bp), *Aat*II (123.598 bp + 51.557 bp + 5.225 bp), and *Pvu*I (176.697 bp + 2.385 bp + 1.298 bp) are exactly as predicted.


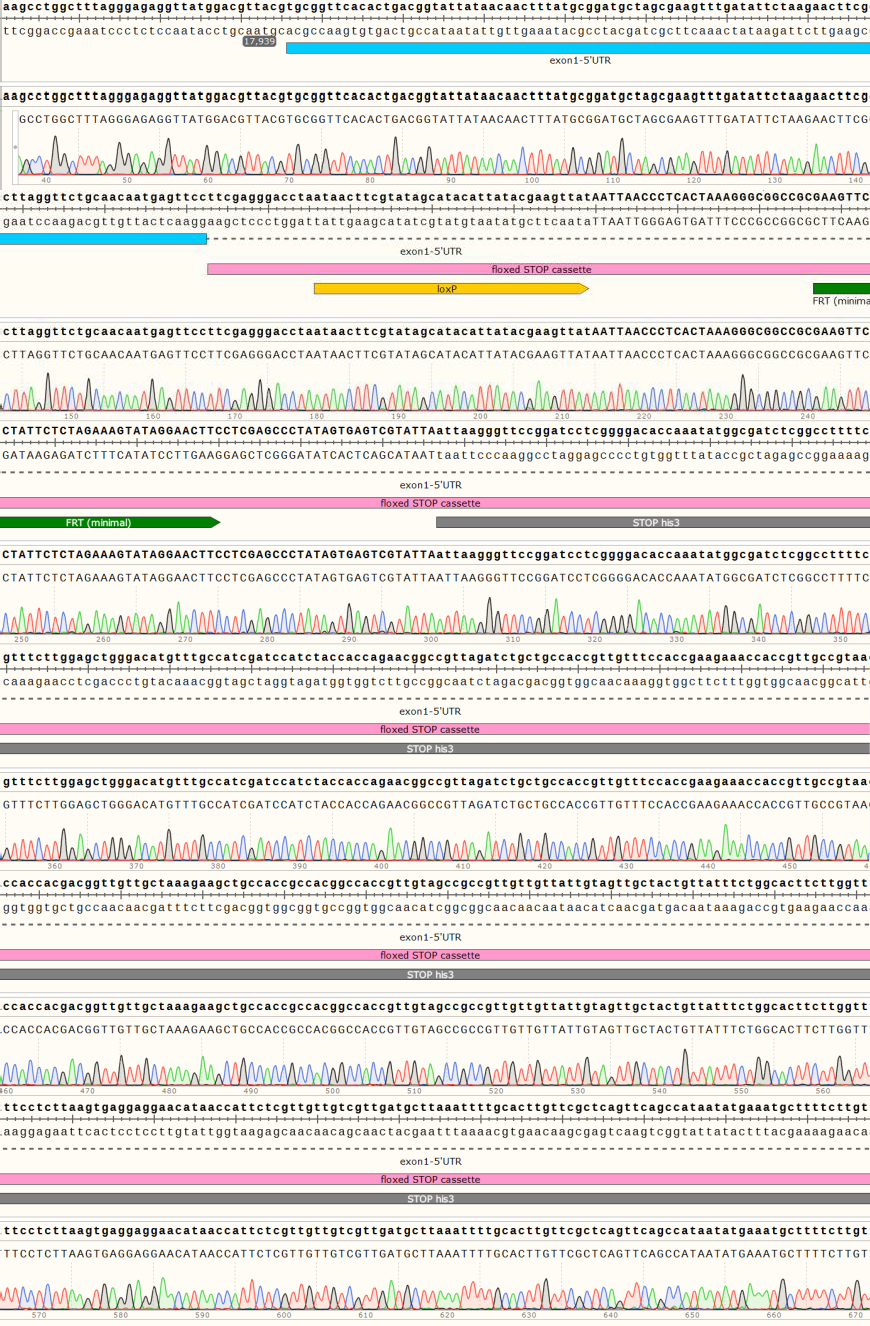

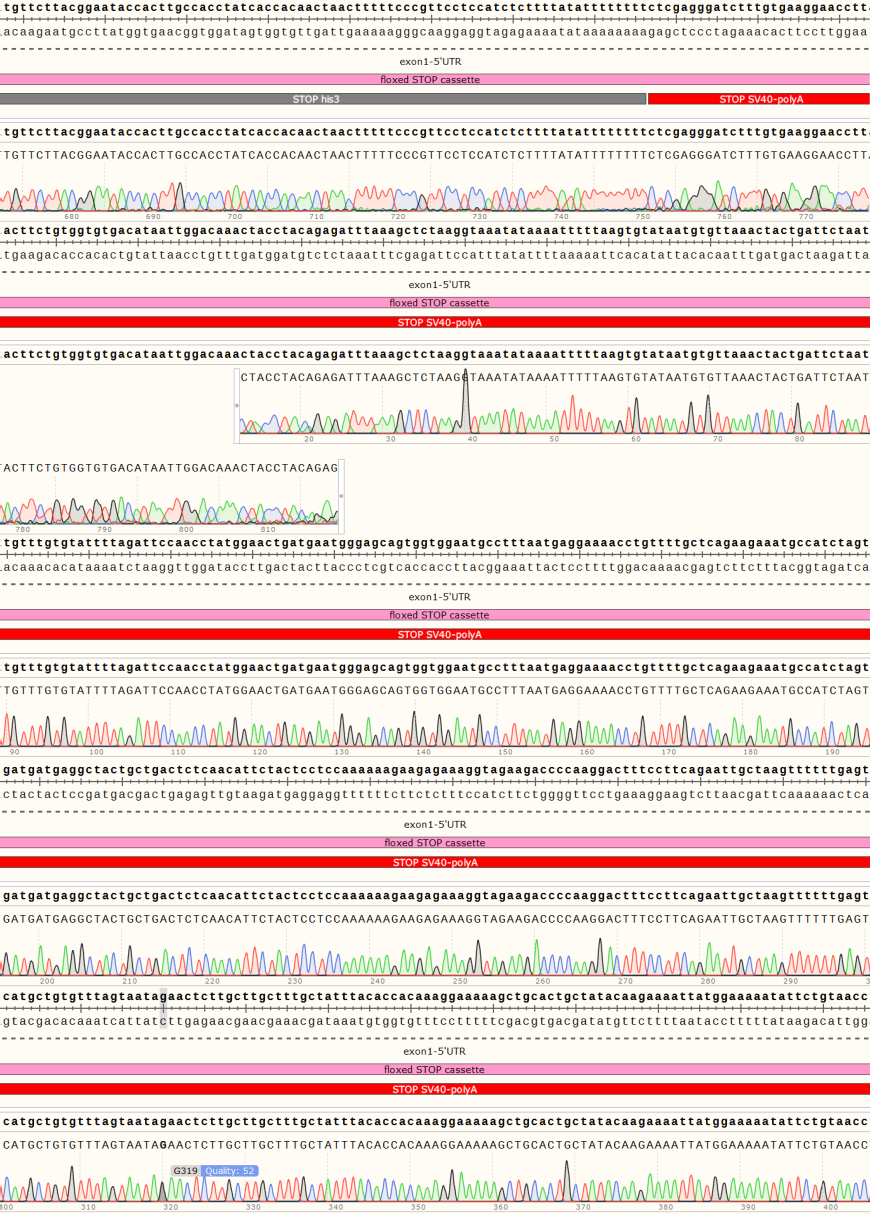


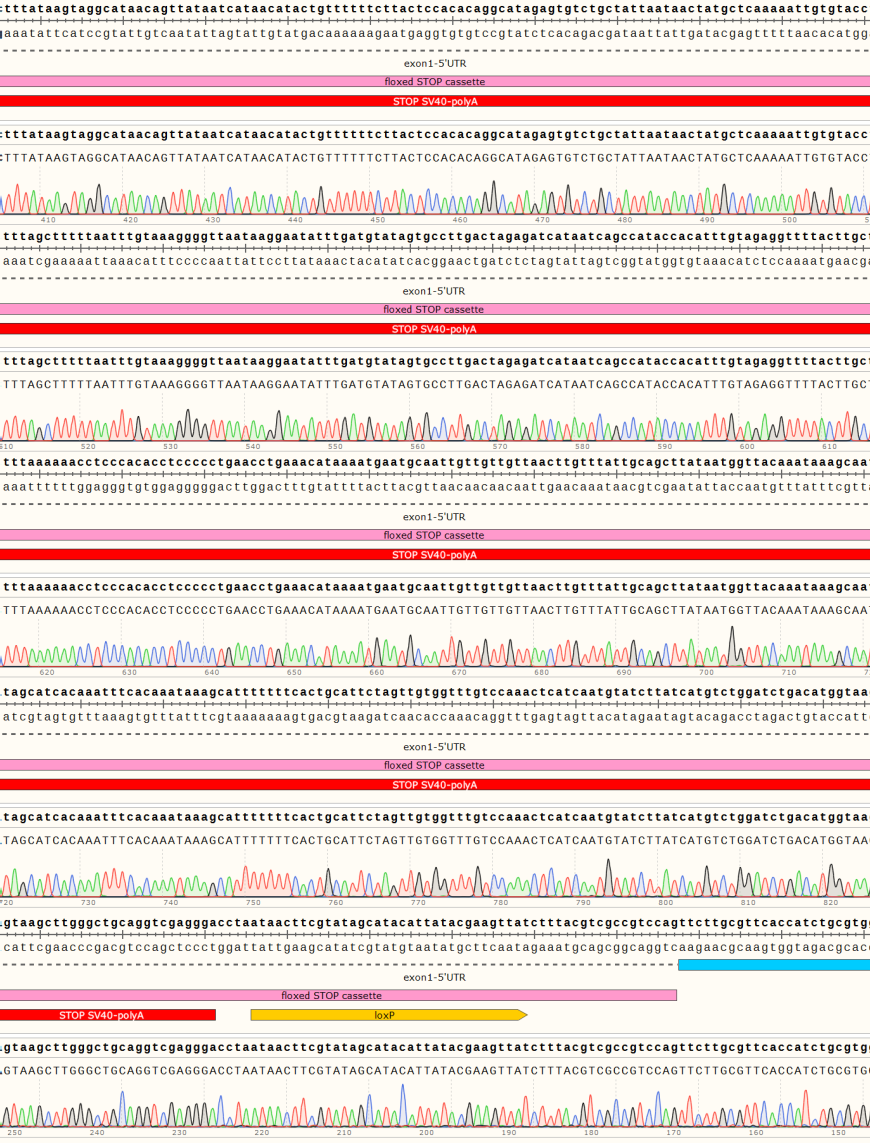

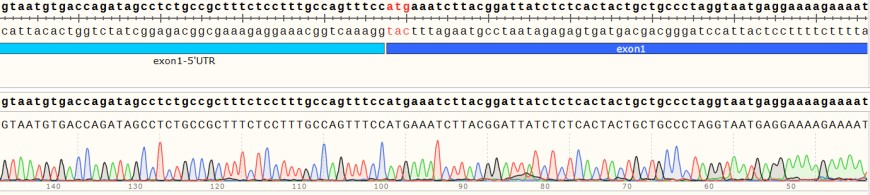


**Supplemental Figure 3:** Sequence of the modified BAC clone (insertion of the floxed STOP cassette).


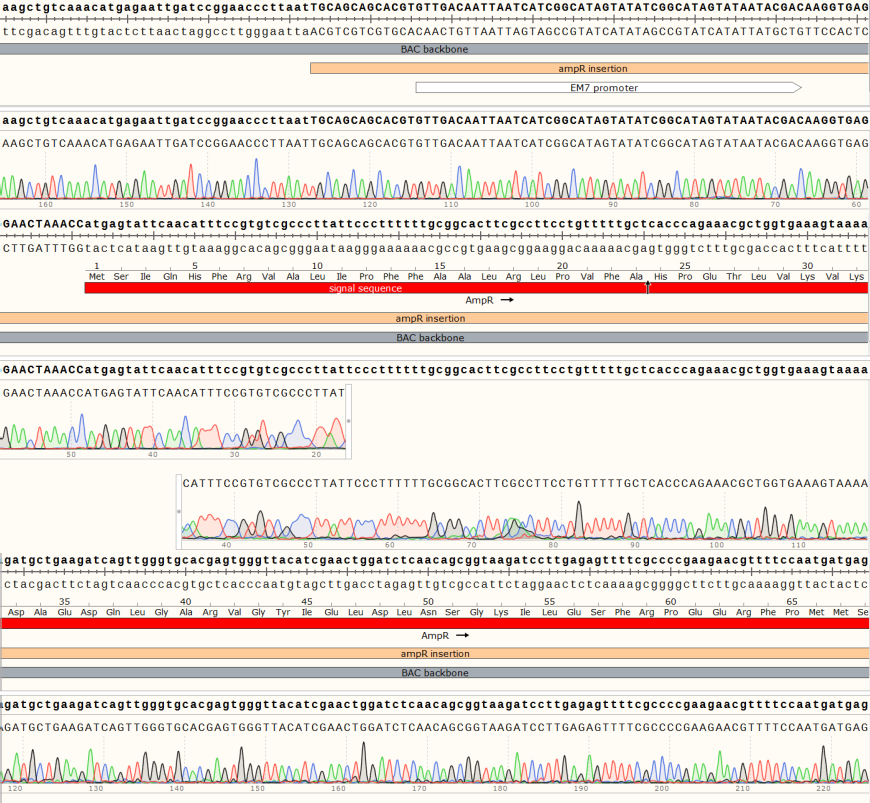


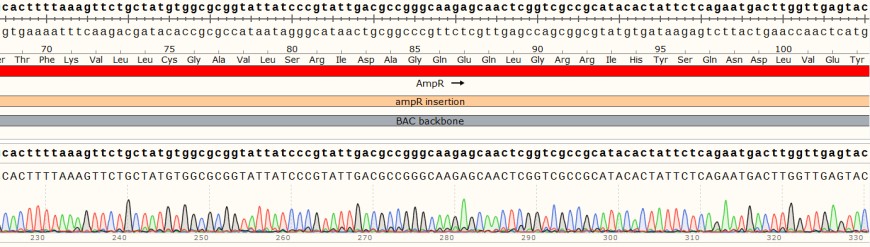

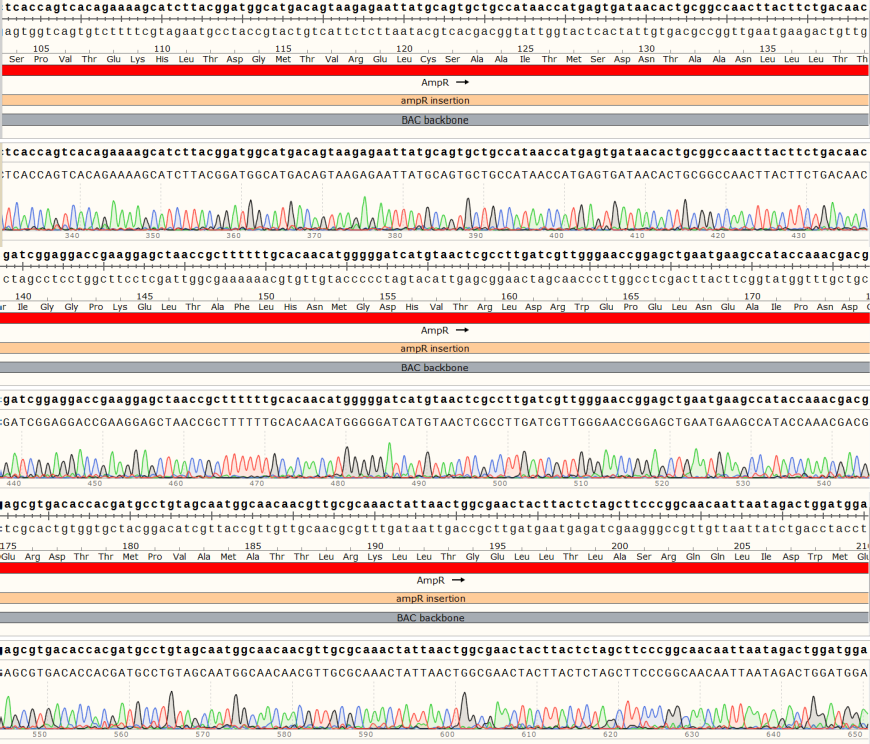


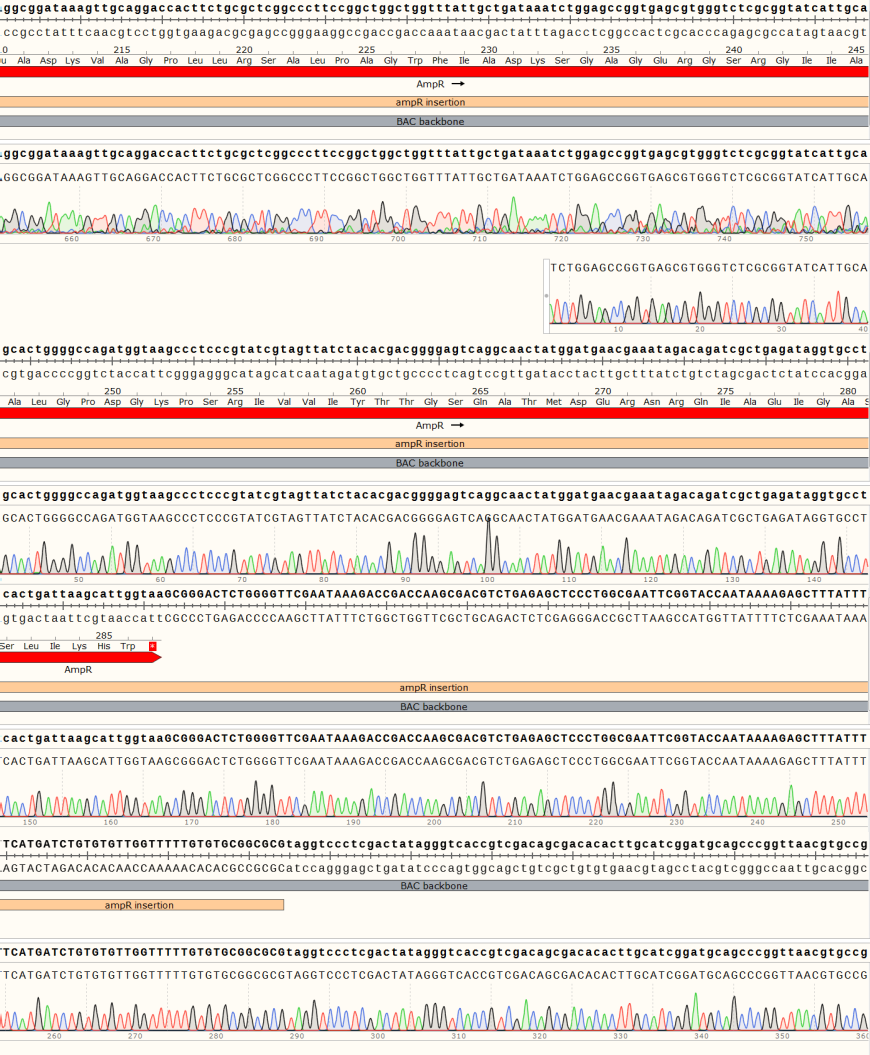
**Supplemental Figure 4:** Sequence of the modified BAC (removal of the loxP in the BAC backbone by ampR): The BAC backbone is indicated by a grey bar below the sequence data; the inserted ampR cassette tan coloured bar.


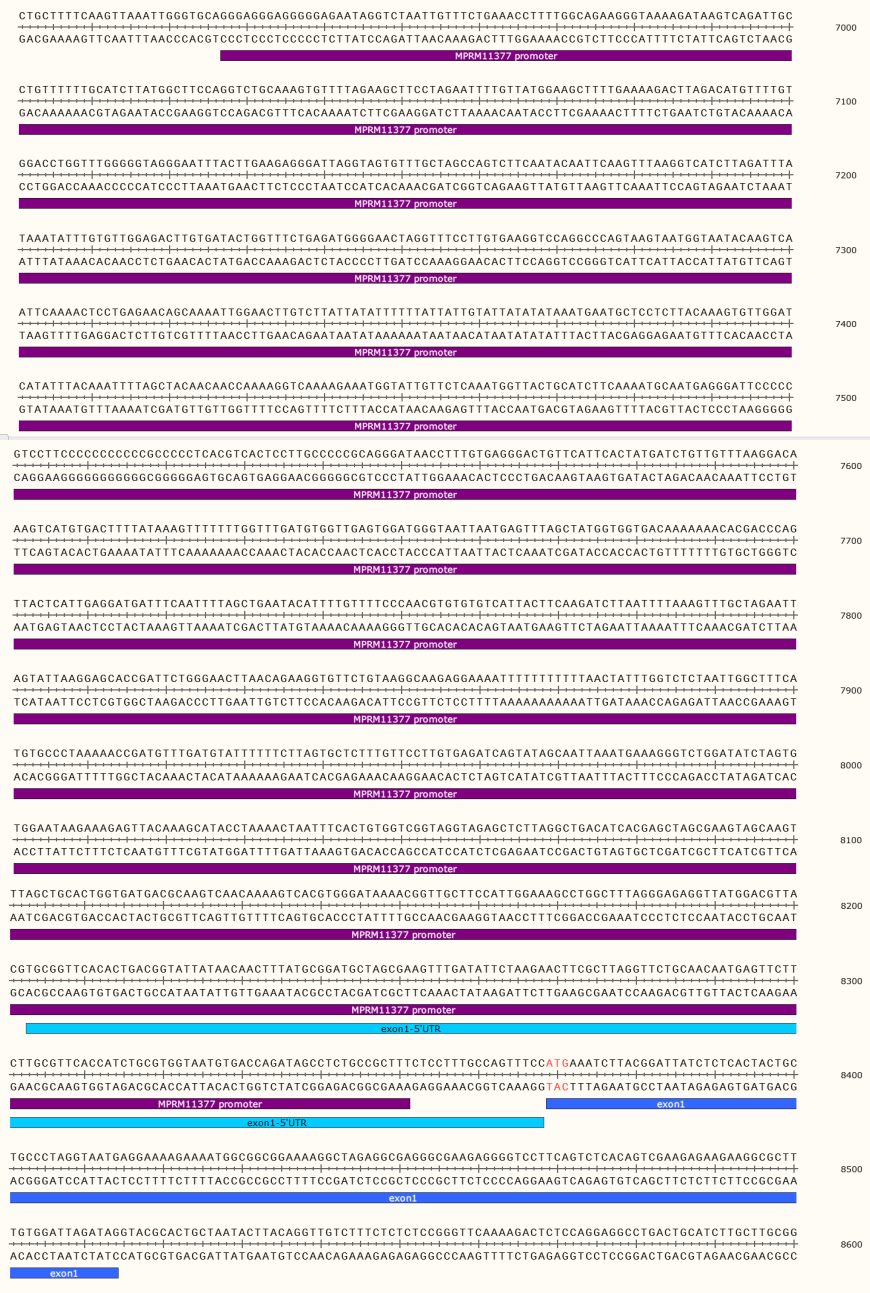
**Supplemental Figure 5:** Sequence of the mouse promoter (MPRM) 11377 (Gene Bridges unique identifier).


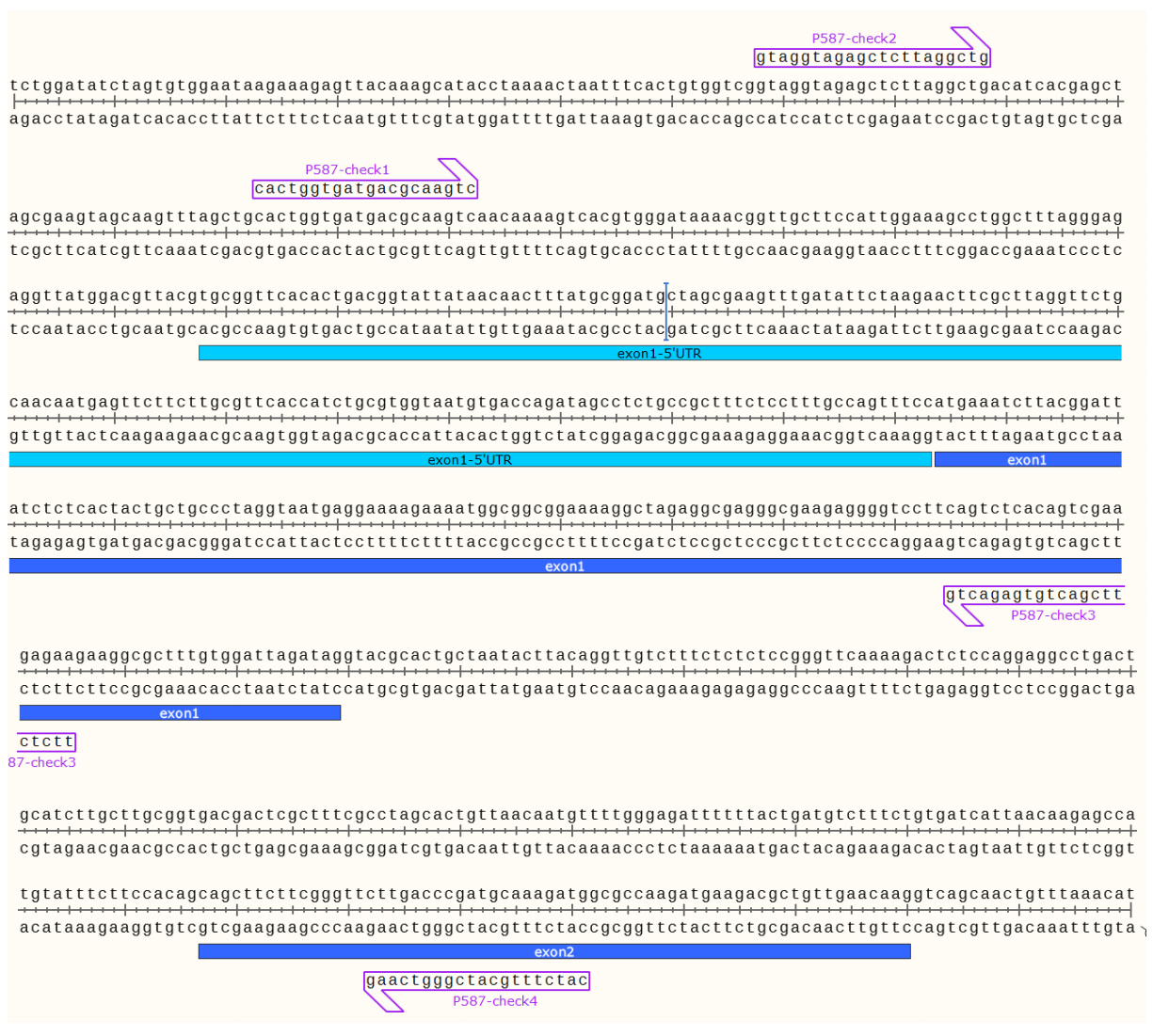

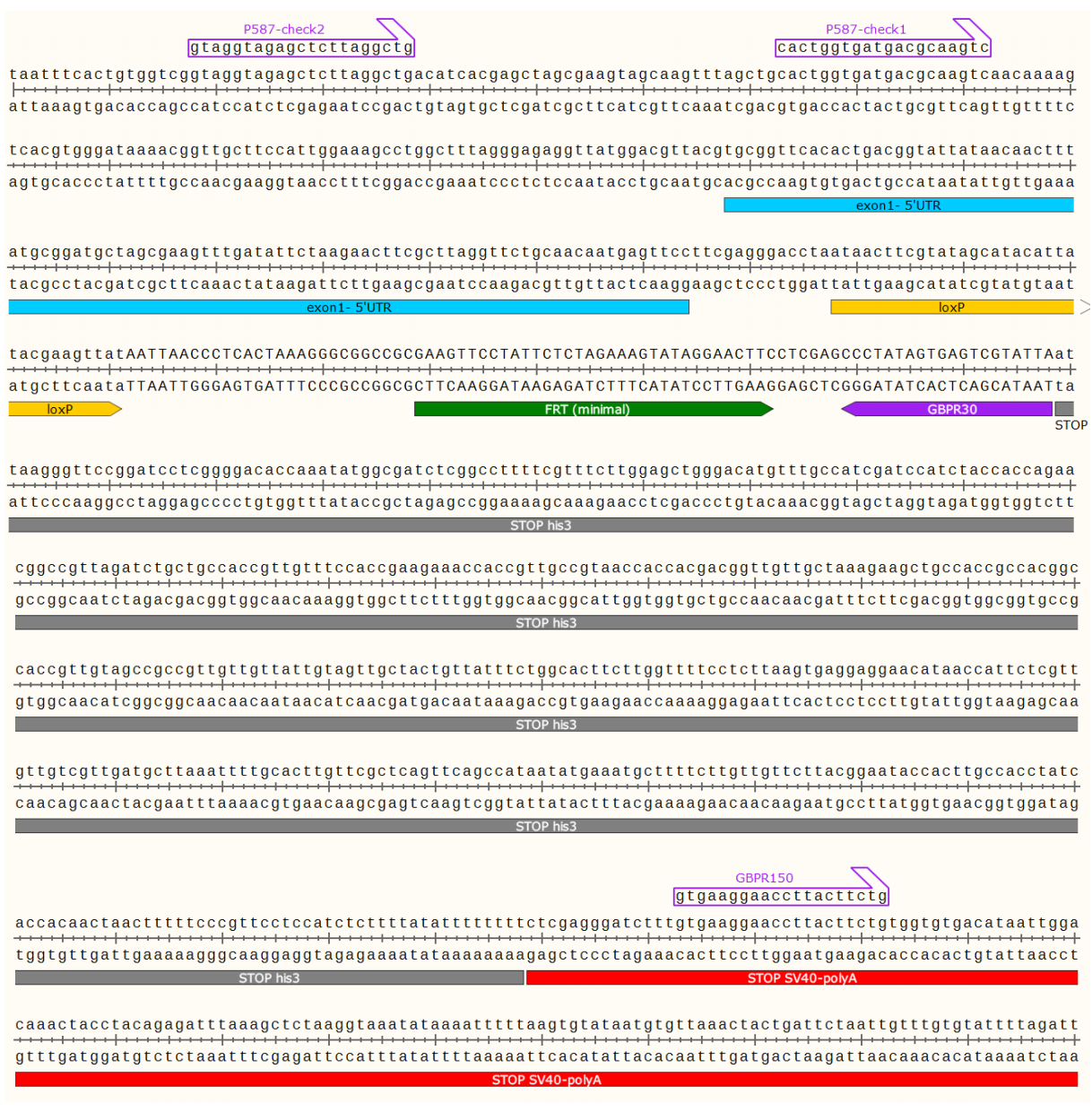

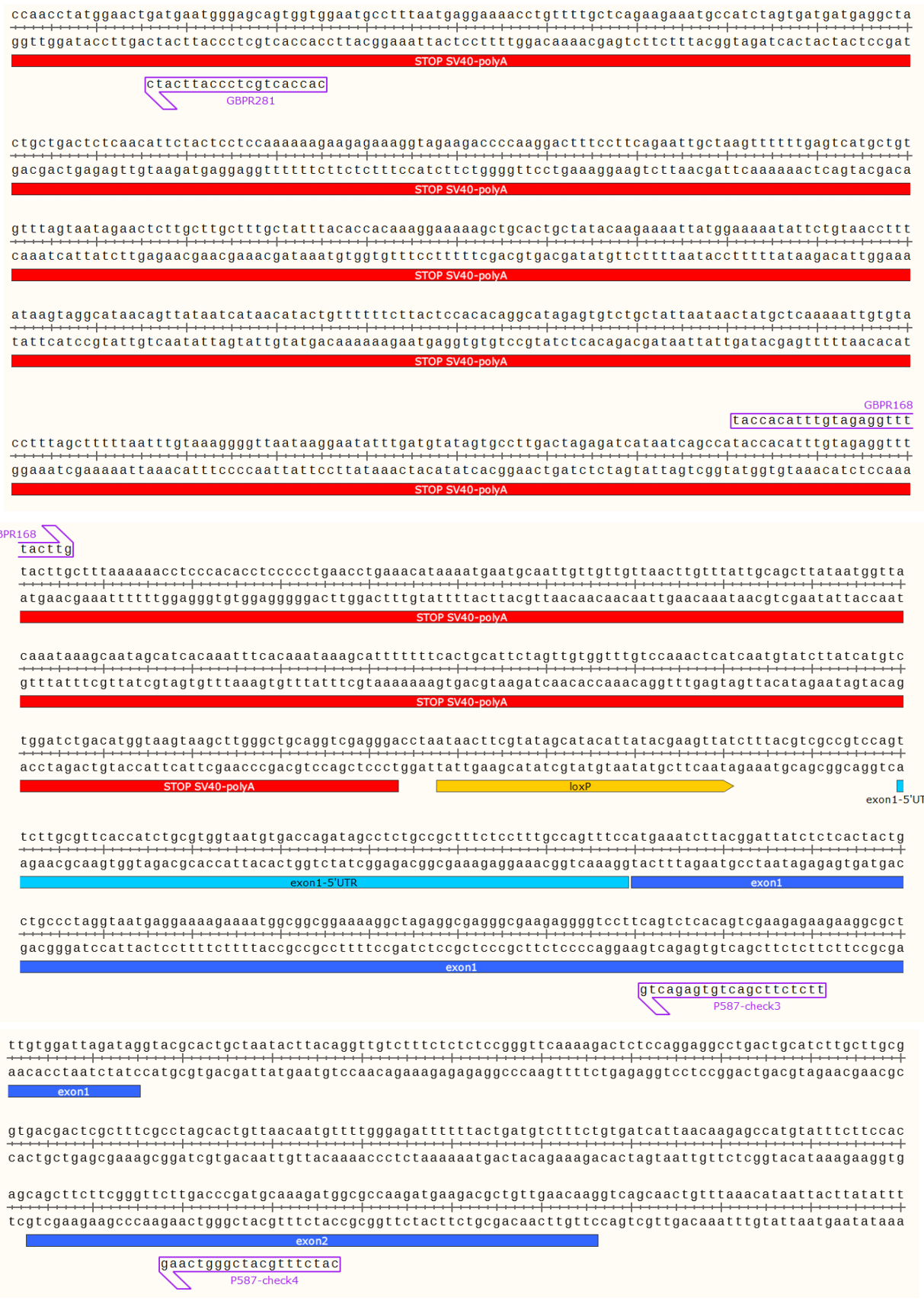


**Supplemental Figure 6:** Polymerase chain reaction primer screening for the wildtype genomic locus, P587 – check1 – check4, and the modified locus, GBPR150, 281, and 168.


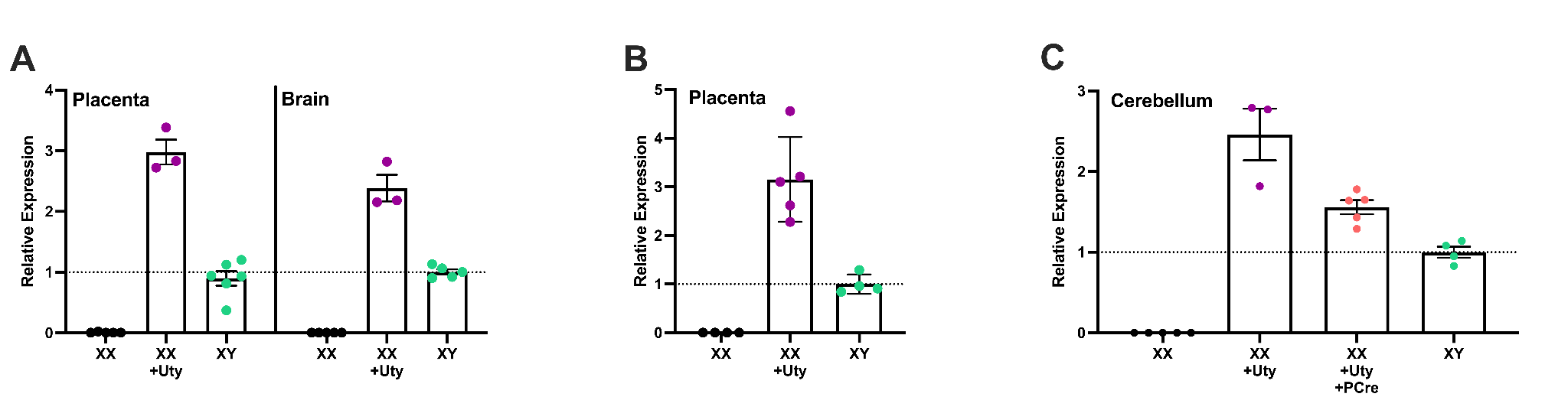


**Supplemental Figure 7**. Relative Uty mRNA expression in A) E18.5 placenta and brain from XX + *PCre* females, XX + *Uty + PCre* females, and XY + *PCre* males (n = 3 – 6), B) E18.5 placenta from XX + *Sim1Cre* females, XY +*Sim1Cre* males, and XX *+Uty* +*Sim1Cre* females (n = 4 – 5), and C) in adult cerebellum from XX females, XX +*Uty* females, XX +*Uty* +*PCre* females, and XY males (n = 3 – 5).


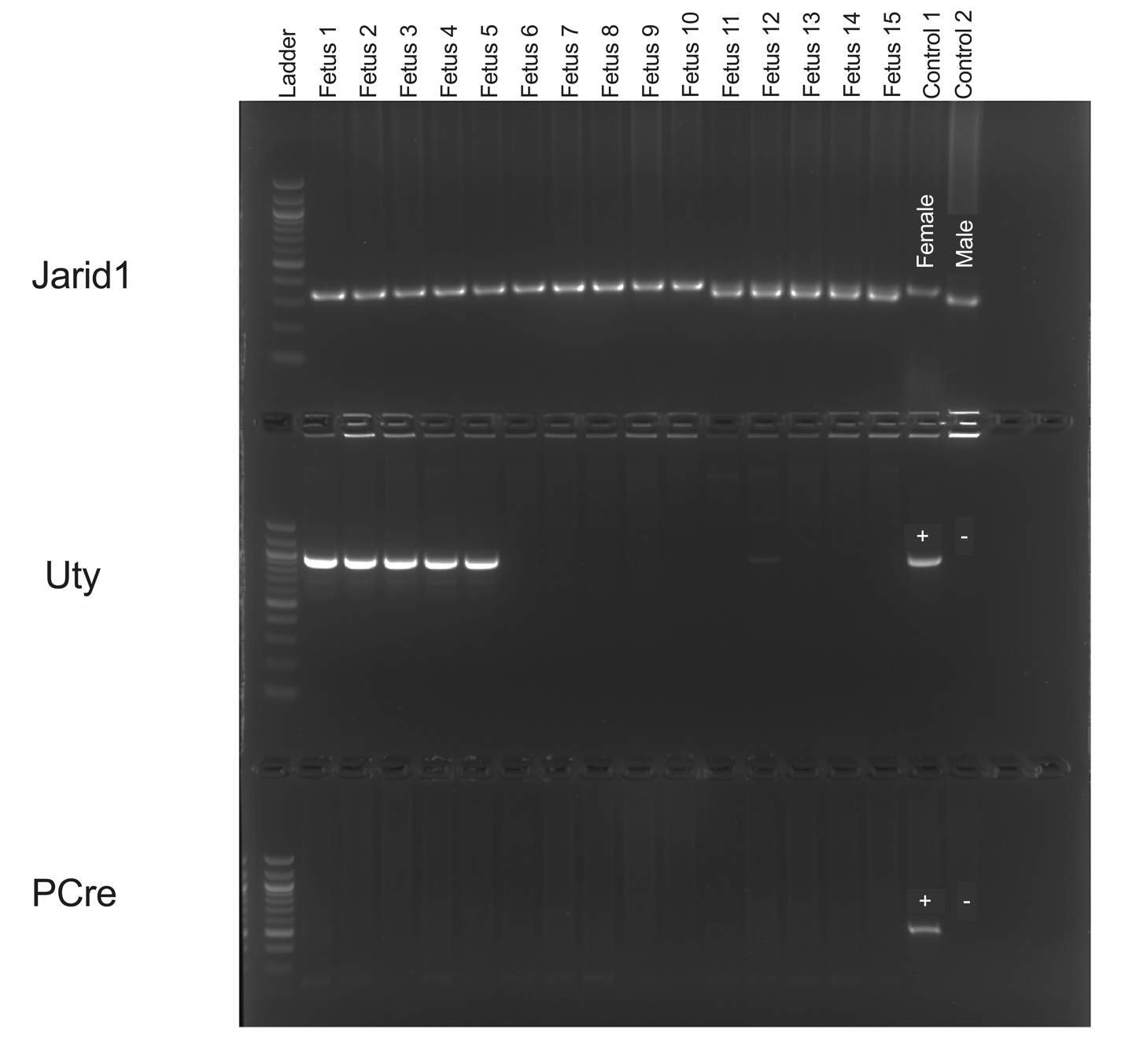


**Supplemental Figure 8.** Image of agarose gel confirming fetal genotype using DNA extracted from fetal tails and amplified for *Jarid1*, *Uty*, and *PCre* (n = 5).

**
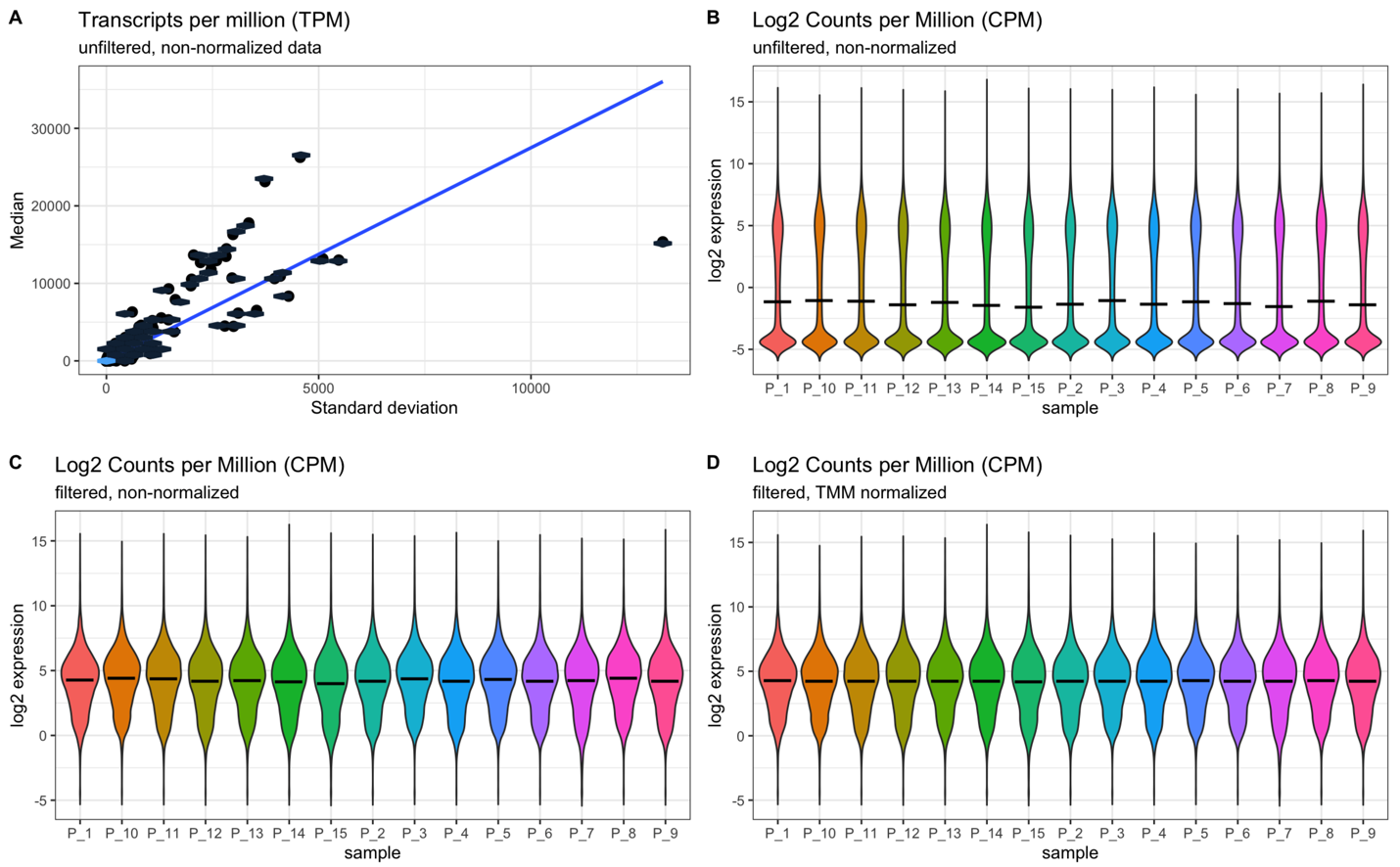
**

**Supplemental Figure 9.** Placenta RNA-Seq data showing **A)** the correlation between median and standard deviation for unfiltered, non-normalized transcripts per million data, and the distributions for Log2 counts per million **B)** unfiltered, non-normalized, **C)** filtered, non-normalized, and **D)** filtered, trimmed mean of M values (TMM) normalized.


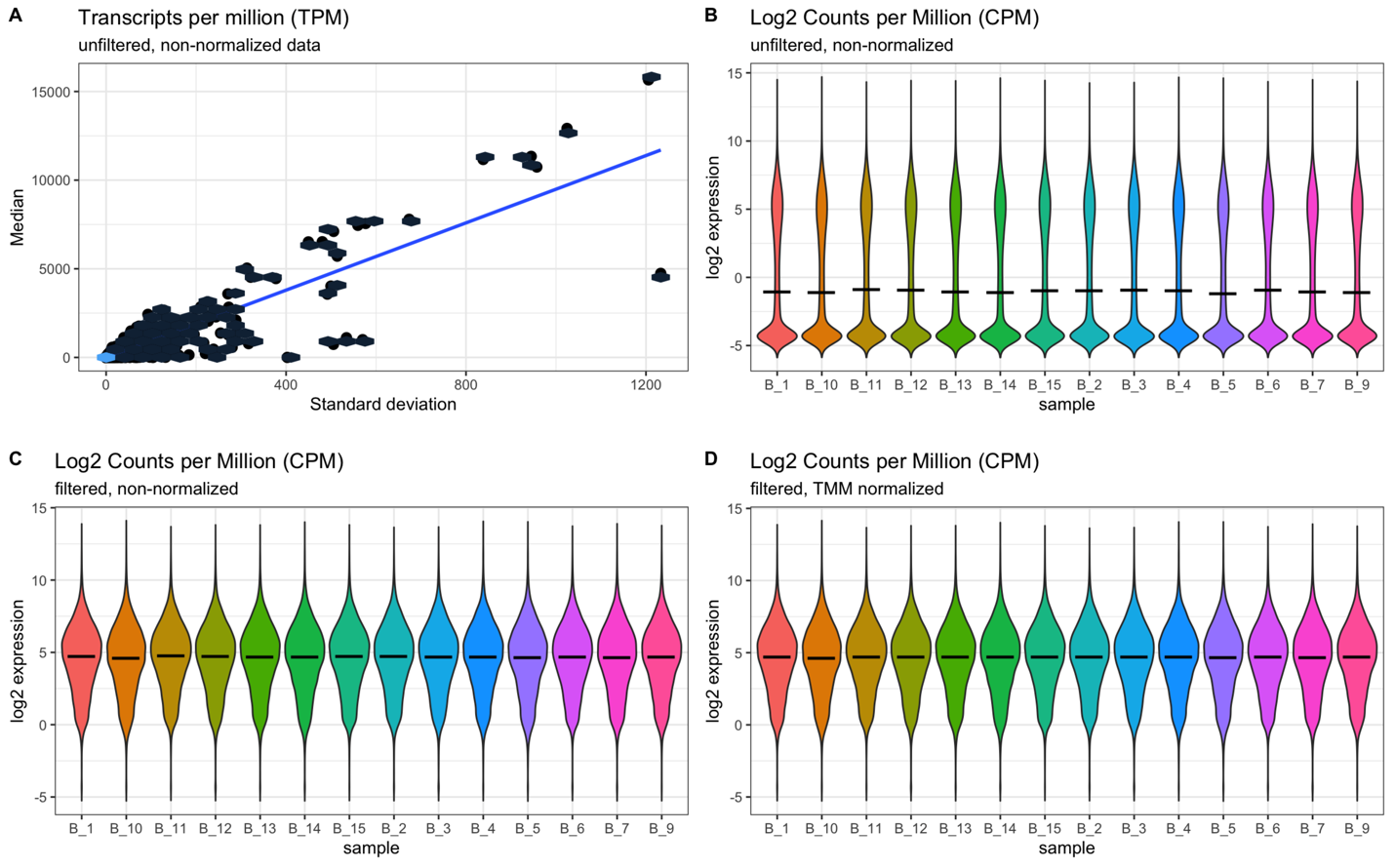


**Supplemental Figure 10.** Hypothalamus RNA-Seq data showing **A)** the correlation between median and standard deviation for unfiltered, non-normalized transcripts per million data, and the distributions for Log2 counts per million **B)** unfiltered, non-normalized, **C)** filtered, non-normalized, and **D)** filtered, trimmed mean of M values (TMM) normalized.

**
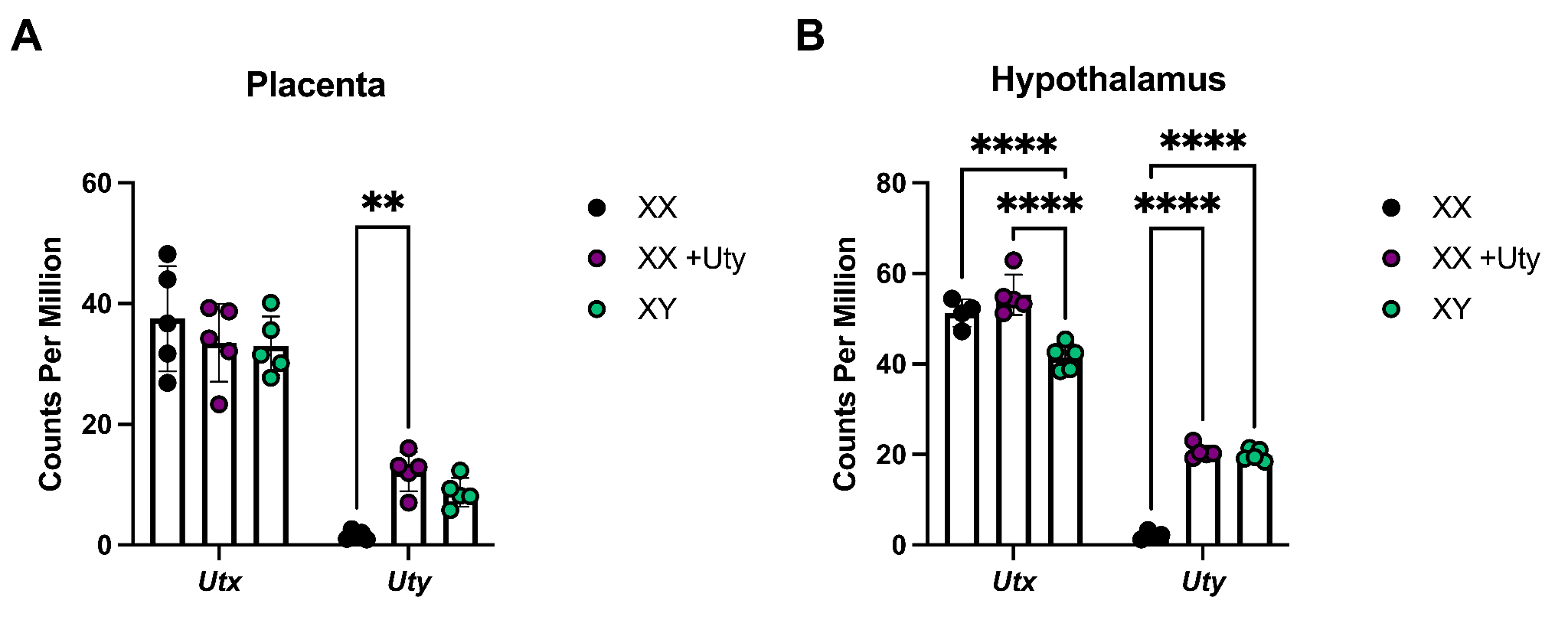
Supplemental Figure 11.** *Utx* and *Uty* counts per million unfiltered, non-normalized as determined by RNA-Sequencing of the **A)** placenta and **B)** hypothalamus (n = 4-5); 2-Way ANOVA, ** p < 0.01, **** p < 0.0001.

**
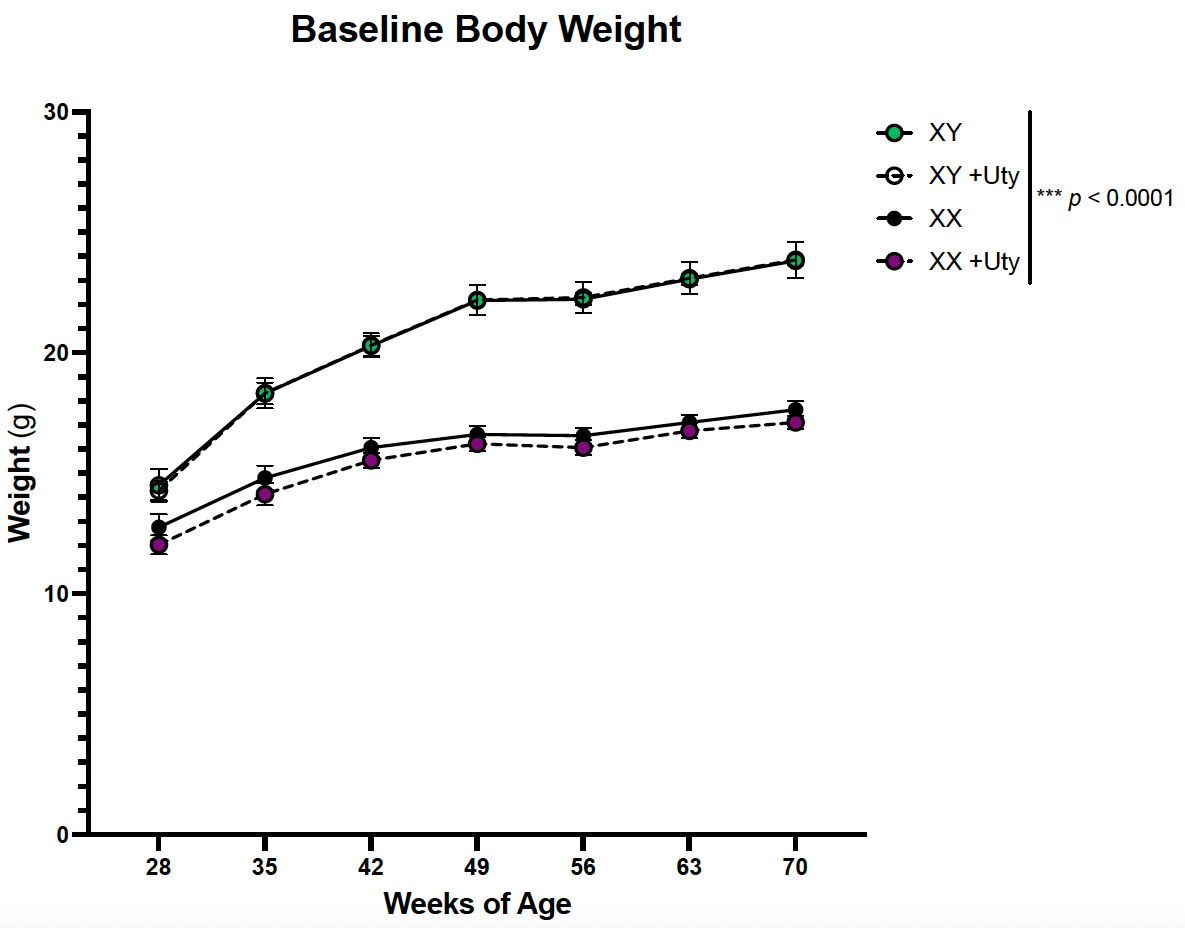
**

**Supplemental Figure 12. XY +Uty animals show no significant change in weight compared to XY males throughout the duration of the study.** Body weights were collected weekly from PN28-70. A significant effect of genotype was observed for baseline body weight measurements (F (2,29) = 63.4, p = 0.0001; η^2^ = 0.81), with XY being consistently heavier than XX +Uty (PN28, p = 0.02; PN35 – PN70, p ≤ 0.0001) and XX (PN35, p = 0.0002; PN42 – 70, p ≤ 0.0001), and XY +Uty consistently heavier than XX +Uty (PN28, p = 0.0006; PN35 – PN70, p ≤ 0.0001) and XX (PN28, p = 0.04; PN35 – PN70, p ≤ 0.0001). There was no significant effect of Uty-Tg on XX +Uty bodyweight compared to XX, or for XY +Uty bodyweight compared to XY (n = 8-12).
